# Lymphoid and CXCR4 Cell Targeted Lipid Nanoparticles Facilitate HIV‐1 Proviral DNA Excision

**DOI:** 10.1002/adhm.202501190

**Published:** 2025-07-14

**Authors:** Sudipta Panja, Lubaba A. Zaman, Chen Zhang, Milankumar Patel, Santhi Gorantla, Prasanta K. Dash, Howard E. Gendelman

**Affiliations:** ^1^ Department of Pharmacology and Experimental Neuroscience University of Nebraska Medical Center Omaha NE 68198 USA

**Keywords:** CRISPR, excision of HIV‐1 DNA, HIV‐1 infection, lipid nanoparticles, targeted mRNA delivery

## Abstract

Advancements in antiretroviral therapy (ART) enable those living with the human immunodeficiency virus type one (HIV‐1) to lead longer, healthier lives free from disease comorbidities. However, lifelong ART poses challenges. These include social stigma, medication costs, drug accessibility, mental health, and drug‐related toxicities. Moreover, ART does not eliminate latent HIV‐1 DNA. Viral persistence in tissue and cell reservoirs results in viral rebound after ART interruption. New strategies are required to achieve a functional HIV‐1 cure. To excise latent HIV‐1, C‐X‐C motif chemokine receptor 4 (CXCR4) ligand‐decorated lymphoid tissue‐targeting lipid nanoparticles (LNPs) for CRISPR‐Cas9/gRNA delivery are developed. These LNPs enhance mRNA translation and demonstrate CXCR4‐mediated improved uptake to eliminate HIV‐1 DNA in infected CD4+ T cells. LNPs also facilitate targeted drug delivery, achieving HIV‐1 DNA excision in ART‐treated, infected humanized mice. This study emphasizes the potential of tissue and cell‐targeted LNPs for effective HIV‐1 DNA excision.

## Graphical abstract

Advancements in ART improve HIV‐1 patient outcomes but are unable to eliminate latent viral DNA. To address this, CXCR4‐targeted lipid nanoparticles (T‐LNPs) for delivering CRISPR‐Cas9 to excise HIV‐1 DNA in infected cells are developed. These T‐LNPs achieve ≈60% HIV‐1 DNA excision efficacy in blood and splenic tissue, demonstrating promise for functional HIV‐1 cures by targeting viral reservoirs.

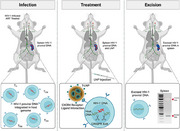

## 1 Introduction

Human immunodeficiency virus type one (HIV‐1) infections affect over 39 million people worldwide.^[^ ^1^ ^]^ While antiretroviral therapy (ART) suppresses viral replication and restores CD4+ T cell counts, HIV‐1 infection remains a significant health concern. Current therapies cannot eliminate the virus from tissue and cellular reservoirs in people living with HIV‐1 (PLWH).^[^ ^2^ ^]^ The latent reservoir of HIV‐1 resides in lymphoid and myeloid cells, which harbor persistent replication‐competent viruses.^[^ ^3^, ^4^ ^]^ Cessation of ART leads to viral rebound.^[^ ^5^ ^]^ Several cure strategies are operative, including immunotherapy, latency reversal agents (LRAs), vaccines, and gene editing therapies.^[^ ^6^ ^]^ Despite progress, no developed strategy can eliminate the virus from its cell and tissue reservoirs.^[^ ^7^, ^8^, ^9^, ^10^, ^11^, ^12^ ^]^ Among the methods developed for viral elimination, clustered regularly interspaced short palindromic repeat (CRISPR)‐Cas9 editing technologies stand out based on their ability to eliminate integrated proviral DNA. This is due to its ability to directly target integrated proviral DNA, independent of the body's immune system.^[^ ^13^, ^14^, ^15^ ^]^ Prior studies reported the development of guide RNAs (gRNAs) to target the LTR‐gag region of the HIV‐1 genome and their delivery by adeno‐associated virus (AAV) vectors.^[^ ^16^ ^]^ The AAV‐based delivery system demonstrated HIV‐1 DNA excision in T‐cell lines, humanized mice (hu mice), and had a favorable safety profile in nonhuman primates.^[^ ^14^, ^17^, ^18^ ^]^

Building on these cumulative findings, Excision BioTherapeutics completed the EBT‐101 clinical trial employing an adeno‐associated virus (AAV)‐based CRISPR‐Cas9 delivery system.^[^ ^19^, ^20^ ^]^ Despite demonstrating safety and highlighting therapeutic CRISPR technology for viral excision, the trial failed to prevent rebound. While AAV offers several delivery advantages, there are drawbacks. This includes vector immunogenicity, limited cargo‐carrying capacities, and inadequate targeting.^[^ ^21^ ^]^ These limitations also restrict the potential for repeated AAV‐CRISPR dosing and may have contributed to the treatment's failure.

LNPs offer advantages over AAV for CRISPR‐Cas9 delivery. For HIV‐1 DNA excision, LNPs can be engineered to target infected lymphoid and myeloid cells, have a high cargo‐carrying capacity, and enable repeated administration.^[^ ^22^, ^23^ ^]^ Moreover, LNPs were used with success in COVID‐19 mRNA vaccines and RNA interference therapies.^[^ ^23^ ^]^ The latter includes the recorded human treatment outcomes for hereditary transthyretin‐mediated amyloidosis.^[^ ^24^ ^]^ Standard LNP formulations consist of four components: ionizable lipid, helper lipid, cholesterol, and PEG‐lipid; they primarily show liver‐selective mRNA translation. Given that lymphoid tissues are a primary reservoir for HIV, we propose redirecting LNPs to the splenic tissues. Incorporating anionic lipids such as 18PA, DOPG, and DOPS into LNP formulations can alter their tropism, directing them from the liver to the spleen.^[^ ^25^ ^]^ The anionic lipids modulate the LNP's surface plasma protein corona and promote lymphoid tissue‐selective mRNA translation through endogenous targeting.^[^ ^25^, ^26^ ^]^ However, high proportions of anionic lipids can reduce mRNA encapsulation efficiency. Therefore, DOPS was selected as the most efficient lipid, as a minimal proportion of added DOPS could effectively promote lymphoid‐tissue selective mRNA translation without compromising encapsulation efficiency.^[^ ^26^ ^]^

To improve cellular targeting in lymphoid tissue, we functionalized LNPs with CXCR4 receptor‐targeting peptide, cyclo(Nal‐Gly‐(D‐Tyr)‐Orn‐Arg‐) (CycPep).^[^ ^27^, ^28^ ^]^ The CXCR4 ligand facilitates HIV‐1 infected cell targeting, as it serves as a co‐receptor for HIV entry into CD4+ T cells. The CXCR4 receptor is expressed in CD4+ T cells within lymphoid tissues, as well as in hematopoietic and endothelial stem cells in the bone marrow.^[^ ^27^, ^28^ ^]^ This dual approach is an advancement towards the development of targeted therapy for HIV‐1 tissue and cell reservoirs. Unlike the LTR‐gag‐based gRNAs, encapsulating these LNPs with our TatD and TatE CRISPR gRNAs may enhance excision efficacy by targeting multiple viral exons (gp41, tat, and rev) at highly conserved sites across more than 4004 HIV‐1 consensus sequences.^[^ ^29^ ^]^ Therefore, these LNPs would enable precise targeting of CXCR4‐expressing CD4+ T cells in the lymphoid tissues to excise HIV‐1 DNA.

Herein, lymphoid tissue‐ and CXCR4‐receptor‐expressing cell‐targeted LNPs were created to facilitate HIV‐1 DNA excision. Upon systemic administration, the DOPS lipid component enables these LNPs to selectively accumulate in lymphoid tissue through endogenous targeting mechanism. Once in the lymphoid tissue, the CycPep ligand on the LNP surface binds to CXCR4 receptors expressed on the CD4+ T cells and delivers the CRISPR‐Cas9/gRNA system directly to the targeted cell. This system efficiently targets and excises the HIV‐1 genome. The success of this LNP formulation represents a significant advancement in developing targeted therapies for the clearance of latent HIV‐1 proviral DNA, bringing us closer to a potential viral cure.

## 2 Results and Discussion

### 2.1 Synthesis of CXCR4 Peptide‐Conjugated PEG Lipid and Formulation of LNPs

**Scheme 1:**
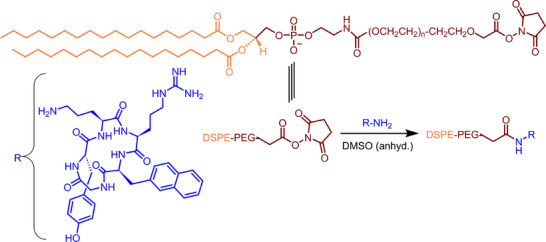
Schematic illustration of the synthesis of DSPE‐PEG‐CycPep via an activated ester‐amine coupling reaction.

The CXCR4 receptor was selected as a target to access HIV‐1‐infected cells. This selection is based on its role as a co‐receptor for HIV‐1 infection of CD4+ T cells and is predominantly expressed in lymphoid tissues and bone marrow.^[^ ^30^ ^]^ To create CXCR4‐receptor‐targeted LNPs, we selected CycPep as a targeting ligand.^[^ ^27^, ^28^ ^]^ This selection is based on CycPep's proven potential as a particular CXCR4 receptor‐targeting ligand. The free amine group (‐NH2) of the ornithine unit in CycPep was conjugated with PEGylated lipid (DSPE‐PEG‐NHS) through an activated ester–amine coupling reaction (**Scheme** **1**).^[^ ^31^, ^32^, ^33^ ^]^

**Figure 1:**
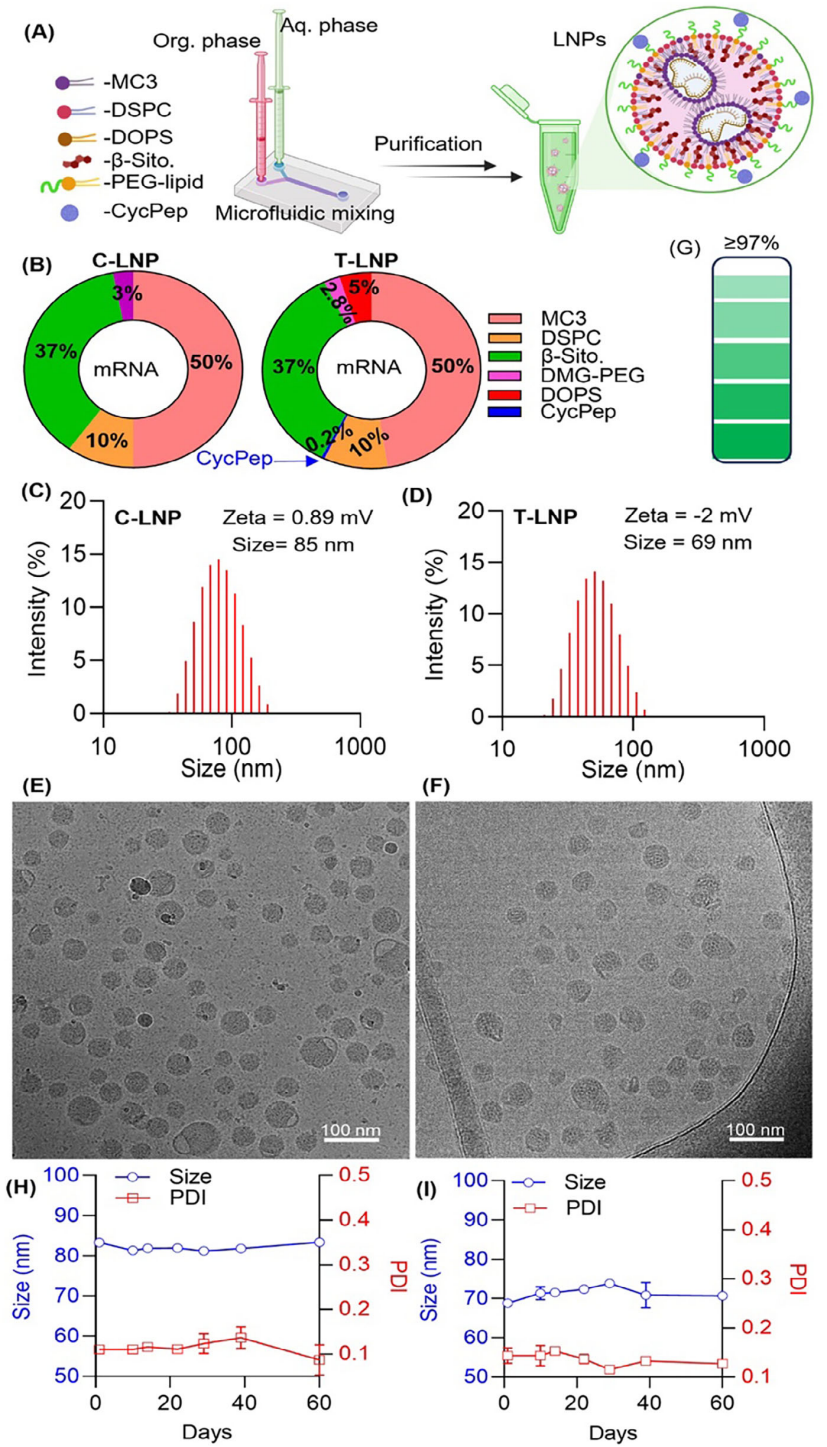
LNP design and characterization. A) Schematic representation of LNP formulation by microfluidic mixing and subsequent purification. Created with BioRender. B) Donut charts showing the molar composition of the lipids used to formulate C‐LNP and T‐LNP. C,D) Size distribution of C‐LNP and T‐LNP determined by dynamic light scattering (DLS). E,F) Cryo‐TEM images of LNPs. G) mRNA encapsulation efficiency of LNPs, evaluated using the RiboGreen assay, indicates >97% encapsulation for both formulations. H,I) The long‐term stability of LNPs was assessed through size and polydispersity index (PDI) measurements (*n* = 3), showing no significant changes.

The successful conjugation of CycPep with DSPE‐PEG‐NHS resulted in the synthesis of DSPE‐PEG‐CycPep, confirmed by ^1^H NMR and mass spectrometry analysis (Figures S1,S2, Supporting Information). In the subsequent step, LNPs were prepared by chaotic microfluidic mixing of the lipid and aqueous phases in a 1:3 volume ratio (**Figure** **1**A). The control LNP (C‐LNP) was formulated with a lipid phase consisting of ionizable lipid (MC3, 50 mol%), helper lipid (DSPC, 10 mol%), β‐sitosterol (37 mol%), and PEG‐lipid (DMG‐PEG, 3 mol%) in ethanol. The aqueous phase (pH 4.5) contained mRNA (Figure 1B). For the targeted LNP (T‐LNP), 0.2 mol% of DMG‐PEG was replaced with DSPE‐PEG‐CycPep, and 5 mol% of DOPS (relative to total lipid mol content) was incorporated into the lipid phase. The aqueous phase remained unchanged. LNPs containing DSPE‐PEG‐CycPep or DOPS alone were prepared as additional controls for the subsequent experiments. DOPS was selected based on its ability to enhance mRNA translation selectively in the lymphoid tissues.^[^ ^26^ ^]^ Therefore, DOPS assists the T‐LNP in reaching the lymphoid tissue, and CycPep further increases the targeting efficacy by selectively binding to CXCR4‐expressing cells inside the targeted tissue. Following microfluidization, the LNPs were dialyzed against PBS at 4 °C for 18 h to exchange the buffer to pH 7.4. Following formulation, the size, dispersity, and surface charge of the LNPs were assessed by dynamic light scattering (DLS). The C‐LNP exhibited an average hydrodynamic diameter of 69 nm, while the T‐LNP displayed a size of 85 nm (Figure 1C,D). Both formulations demonstrated unimodal size distributions with a polydispersity index (PDI) of ≤ 0.15. Cryogenic transmission electron microscopy (Cryo‐TEM) images revealed that the LNPs were spherical (Figure 1E,F). The LNP's zeta potential ranged from 0.89 to −2 mV, indicating a nearly neutral surface charge. However, a near‐neutral zeta potential does not inherently lead to aggregation, especially in the presence of PEG lipids. PEG contributes significantly to the stability of LNPs by providing steric stabilization, improving dispersibility, and imparting stealth properties.^[^ ^34^ ^]^ PEG forms a hydration shell around the LNPs, preventing aggregation and enhancing dispersion.^[^ ^35^ ^]^ The apparent pKa of the LNPs was determined using a 2‐(p‐toluidino)‐6‐naphthalenesulfonic acid (TNS) assay across buffers with pH values ranging from 2 to 12. The apparent pKa values of T‐LNP and C‐LNP were determined to be 6.37 and 6.20, respectively, indicating their suitability for endosomal escape and efficient mRNA delivery (Figure S3A,B, Supporting Information).^[^ ^36^ ^]^ The mRNA encapsulation efficiency of the LNPs was ≥97% (Figure 1G). The long‐term stability of a formulation is crucial for evaluating its time‐dependent usability, safety, and efficacy. LNPs were stored at 4 °C to assess this, and their size, polydispersity, and mRNA encapsulation efficiency were monitored. Over two months, no significant changes were observed in any of these characteristics, indicating their usability at least two months after formulation (Figure 1H,I; Figure S3C,D, Supporting Information).

### 2.2 Lymphoid Tissue Targeted mRNA Delivery

**Figure 2:**
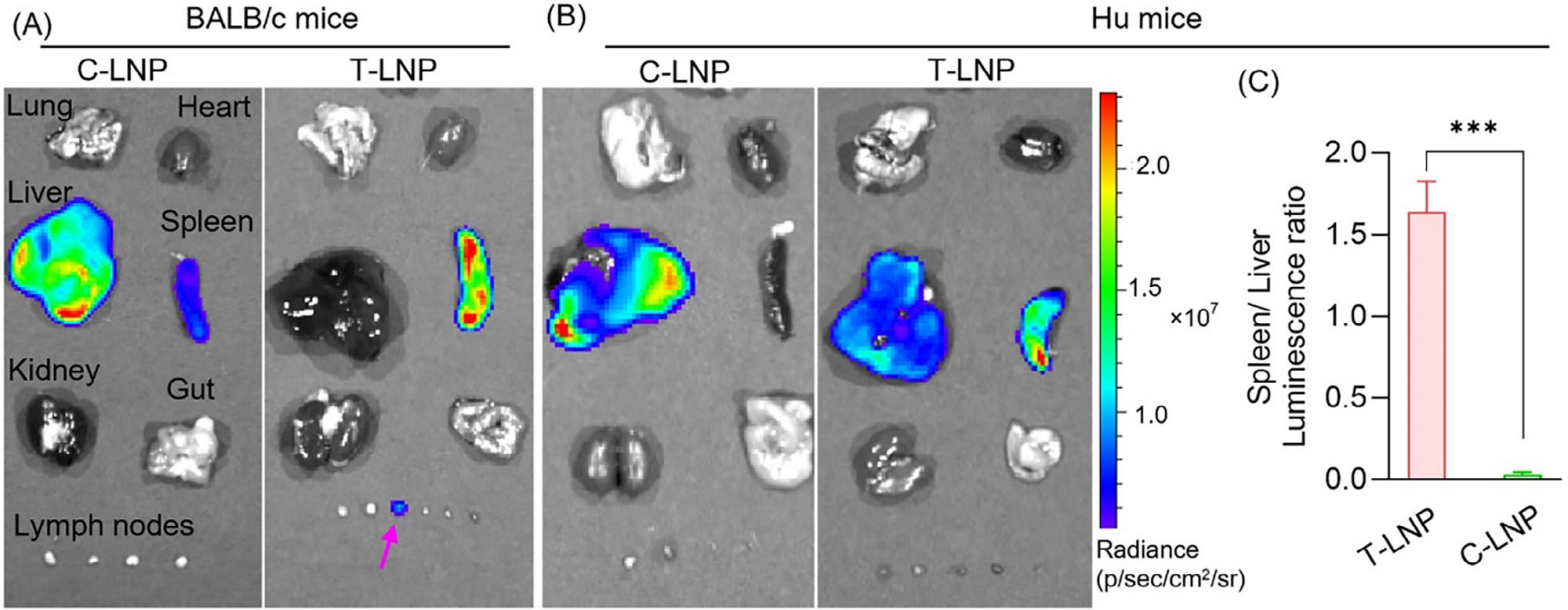
Tissue selective mRNA translation of LNPs. A,B) Tissue‐selective mRNA translation efficiency of LNPs in BALB/c and humanized mice. C‐LNP exhibits liver‐selective mRNA translation, while T‐LNP shows spleen‐selective mRNA translation. C) Spleen‐to‐liver luminescence ratio in humanized mice (*n* = 3), highlighting the spleen tissue selectivity of T‐LNP. The mRNA translation efficiency was evaluated after tail vein administration of LNPs at 0.5 mg kg^−1^ mRNA dose. Luminescence images were acquired 6 h following administration using an in vivo imaging system (IVIS). Data are presented as means ± SD (*n* = 3). ^***^ *p* < 0.001.

Targeted mRNA delivery to HIV reservoirs is crucial for effectively reaching reservoir tissues. Although LNPs have been successfully used in mRNA‐based vaccines and therapeutics, they have not yet been developed for HIV therapies. Most LNPs exhibit liver‐selective mRNA translation due to the formation of plasma protein corona. The apolipoprotein E (ApoE)‐enriched corona enables LNPs to be recognized by the liver's low‐density lipoprotein receptor.^[^ ^37^ ^]^ This drives liver‐selective protein translation, which is known as endogenous targeting.^[^ ^38^ ^]^ As lymphoid tissues are key reservoirs of latent HIV and play a critical role in viral persistence,^[^ ^39^, ^40^ ^]^ we sought to shift LNP tropism from the liver to lymphoid tissues. We tailored the composition of C‐LNPs by incorporating an anionic lipid, DOPS. This modification alters the protein corona by enriching it with β2‐glycoprotein I, facilitating selective mRNA translation in lymphoid tissues.^[^ ^26^, ^41^, ^42^ ^]^ We prepared T‐LNP by adding tissue‐targeting DOPS and cell‐targeting DSPE‐PEG‐CycPep to the C‐LNP's lipid mixture. LNPs were formulated using firefly luciferase (FLuc) mRNA as a reporter gene. The LNPs were administered to BALB/c mice via tail vein injection (mRNA dose: 0.5 mg kg^−1^). Six hours following the injection, D‐luciferin was administered intraperitoneally, and FLuc expression was detected using an in vivo imaging system (IVIS) (**Figure** **2**A). While C‐LNPs primarily exhibited FLuc expression in the liver, T‐LNPs showed increased expression in the spleen and lymph nodes. As we needed to test the LNP efficacy in hu mice, we further conducted the FLuc expression study in these mice. As expected, C‐LNPs predominantly displayed FLuc expression in the liver. However, T‐LNPs showed higher FLuc expression in the spleen (Figure 2B; Figure S4A, Supporting Information). To further assess tissue specificity, we compared spleen‐to‐liver luminescence ratios. T‐LNPs exhibited 1.8 times higher luminescence in the spleen than C‐LNPs, indicating spleen targeted mRNA delivery (Figure 2C). No signals were detected in lymph nodes, reflective of their underdevelopment in the immunocompromised mice.^[^ ^43^ ^]^ To investigate whether DSPE‐PEG‐CycPep or DOPS alone influences tissue tropism, we evaluated the FLuc mRNA translation of LNPs containing only DSPE‐PEG‐CycPep or DOPS. The results revealed that LNPs containing DSPE‐PEG‐CycPep alone could not alter tropism, while the LNPs with DOPS shifted tropism to the spleen (Figure S4B,C, Supporting Information). We combined DSPE‐PEG‐CycPep with DOPS in the T‐LNP formulation to achieve both tissue and cell targeting.

### 2.3 CXCR4‐Cell Targeted LNP Delivery

**Figure 3:**
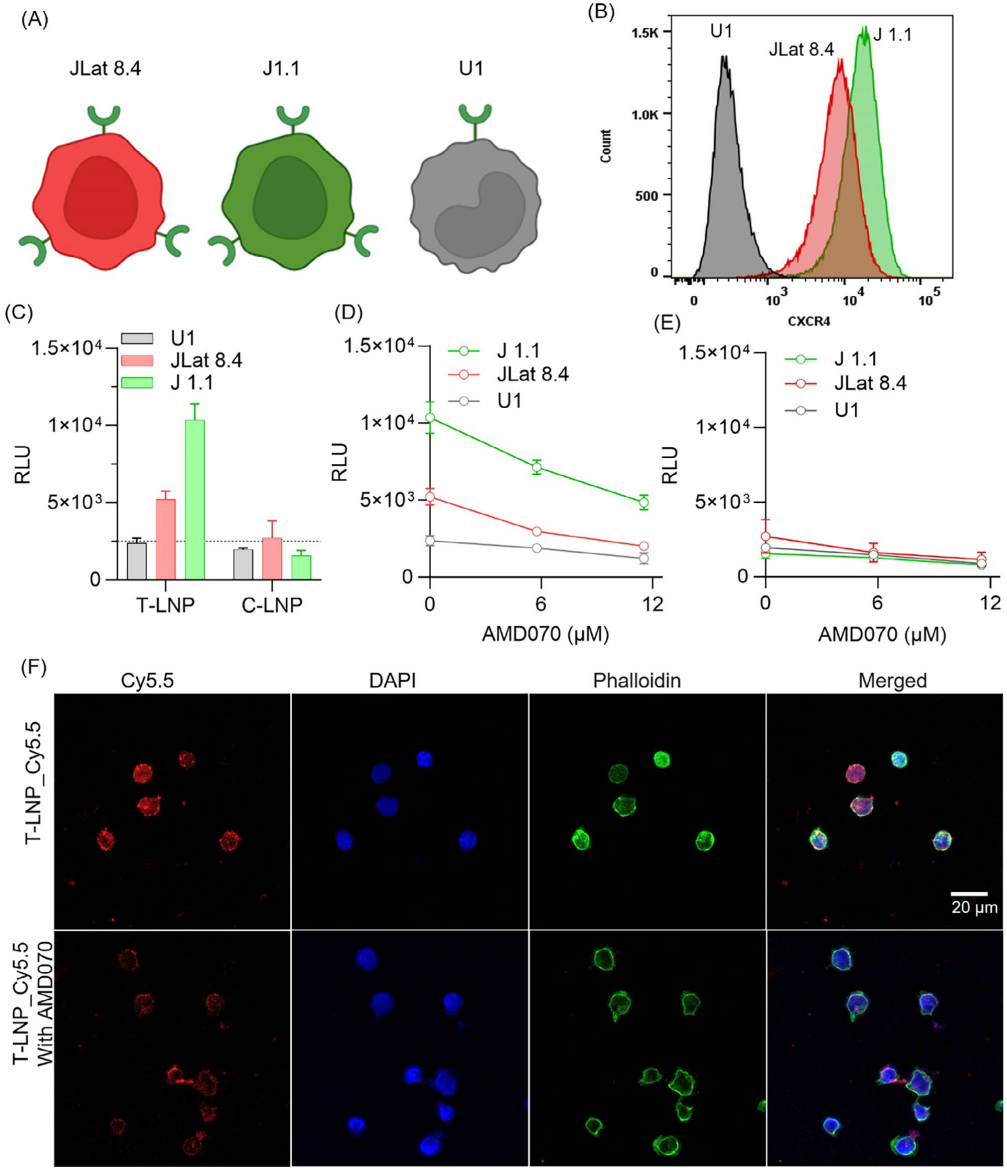
CXCR4‐dependent uptake and mRNA translation. A) Schematic representation of latent HIV‐1‐infected cell lines (lymphocytic JLat 8.4, J1.1, and promonocytic U1) and their respective CXCR4 expression. Created with BioRender. B) Quantification of CXCR4 expression by flow cytometry. J1.1 cells show the highest CXCR4 expression, followed by JLat 8.4 and U1 cells. C) Luciferase mRNA translation efficacy of LNPs across different cell lines. The mRNA translation efficiency of these LNPs was assessed 48 h post‐LNP treatment (at 1 µg/million cells mRNA dose) using the luciferase assay and presented as relative luminescence units (RLU). D,E) Concentration‐dependent CXCR4‐receptor inhibition by AMD070 and its impact on mRNA translation efficiency of T‐LNP and C‐LNP across various cell lines. Data are presented as means ± SD (*n* = 3). F) Effect of AMD070 pretreatment on the cellular uptake of Cy5.5‐labeled T‐LNPs in J1.1 cells. Cells were pretreated with AMD070 for 30 min, followed by treatment with Cy5.5‐labeled T‐LNPs. After 4 h, cells were co‐stained with DAPI (blue) and phalloidin (green) and visualized using confocal microscopy.

To demonstrate cell‐selective mRNA delivery by LNPs, we chose two lymphocytic cell lines, JLat 8.4 and J1.1, both with high levels of CXCR4 expression. Additionally, we included the promonocytic U1 cell line, characterized by low CXCR4 expression (**Figure** **3**A). All three cell lines are commonly used as HIV‐1 latently infected cell models.^[^ ^3^, ^44^ ^]^ The differential expression of CXCR4 receptors across all cell lines was further confirmed by flow cytometry analysis (Figure 3B). The cell lines were treated with LNPs with decreasing concentrations of an equivalent mRNA dose ranging from 16 to 0.5 µg/million cells, and cell viability was assessed at 48 h of post‐treatment using the CellTiter‐Blue assay. The dose of 8 µg/million cells was found to be the maximum safe dose, as it maintained cell viability above 80% (Figure S5, Supporting Information). Based on this, the freshly cultured cells were treated with LNPs containing FLuc mRNA at 1 µg/million cells. The mRNA translation efficiency of these LNPs was assessed 48 h after treatment using the luciferase assay and presented as relative luminescence units (RLU). In comparison to C‐LNPs, T‐LNPs exhibited higher RLU values, indicating enhanced protein translation efficacy (Figure 3C). Notably, JLat 8.4 and J1.1 cells exhibited significantly higher RLU values compared to U1 cells when treated with T‐LNPs. Indeed, this difference closely correlates with their respective CXCR4 expression levels (Figure 3B). To affirm if the CXCR4‐receptormediated this effect, we pretreated the cells with increasing concentrations of AMD070, a CXCR4 antagonist. ^[^ ^45^, ^46^, ^47^ ^]^ Following a safe dose of AMD070 pretreatment (Figure S5C, Supporting Information), T‐LNP treated J1.1 and JLat 8.4 cells showed a gradual decrease in luciferase expression with increasing AMD070 concentrations (Figure 3D). At the same time, C‐LNP treatment exhibited no significant changes (Figure 3E). These results indicate that T‐LNP‐mediated enhanced protein translation was CXCR4 receptor‐dependent. Cy5.5 dye‐labeled T‐LNPs were treated to J1.1 cells with and without the AMD070 pretreatment to validate this observation further. At 4 h post‐treatment, cells were co‐stained with DAPI (blue) for nuclear labeling and phalloidin (green) for cytoskeleton visualization, then visualized under confocal microscopy (Figure 3F). The microscopy images revealed intense red fluorescence in T‐LNP‐treated J1.1 cells, with a substantial decrease in fluorescence intensity following AMD070 pretreatment. This decrease in fluorescence intensity suggests that blocking CXCR4 with AMD070 decreased the T‐LNP uptake and, consequently, FLuc protein expression. Moreover, a certain level of fluorescence intensity was observed even after CXCR4 blocking, indicating that a fraction of the LNPs enter the cells via a CXCR4‐independent pathway.^[^ ^48^ ^]^ However, this fraction is considerably modest, as evidenced by the significant difference in Cy5.5 intensity between AMD070‐pretreated and untreated cells (Figure S5D, Supporting Information). Together, these results confirm the CXCR4‐expressing cell selective uptake and enhanced mRNA translation of T‐LNP.

### 2.4 HIV‐1 DNA Excision Efficacy in Infected T‐Cell Lines

**Figure 4:**
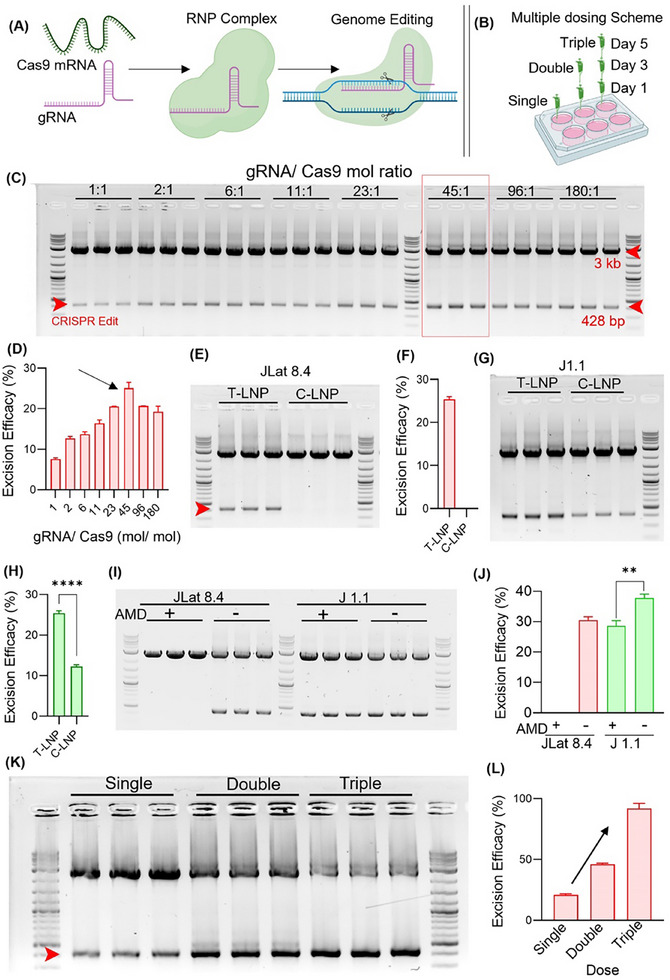
HIV‐1 proviral DNA excision efficacy of CRISPR‐Cas9 LNPs. A,B) Schematic of the CRISPR‐Cas9 gene‐editing process and multiple doasing. Created with BioRender. C) PCR gel electrophoresis image of DNA extracted from JLat 8.4 cells treated with LNPs (1 µg/10⁶ cells) formulated with varying gRNA/m1Ψ‐mCas9 molar ratios. Red arrowheads denote the proviral DNA amplicon (3 kb) and the excised DNA amplicon (428 bp). D) Quantifying HIV‐1 DNA excision efficacy of LNPs formulated with different gRNA/m1Ψ‐Cas9 molar ratios, analyzed using ImageJ and densitometric analysis. E–H) The comparison and quantification of HIV‐1 DNA excision efficacy between T‐LNP and C‐LNP in lymphocytic (E, F) JLat 8.4 and (G, H) J1.1 cell lines. I) Effect of AMD070 pretreatment on the HIV‐1 DNA excision efficacy of T‐LNPs in JLat 8.4 and J1.1 cell lines. J) The differences in excision efficacy following AMD070 treatment were quantified using ImageJ and densitometric analysis. K,L) PCR gel electrophoresis image and quantification of HIV‐1 DNA excision efficacy following multiple doses of LNP treatment in the JLat 8.4 cell line. Data are presented as means ± SD (*n* = 3). ^**^ *p* < 0.01 and ^****^ *p* < 0.0001.

Next, we assessed the CRISPR editing efficacy of these LNPs by encapsulating Cas9 mRNA and gRNAs. Although the successful cytoplasmic translation of Cas9 mRNA (similar to FLuc mRNA) is essential, it alone does not ensure effective gene editing. After translation, the Cas9 protein must form a ribonucleoprotein complex with the gRNA and be protected from degradation.^[^ ^49^ ^]^ This complex then needs to translocate into the nucleus for targeted CRISPR editing (**Figure** **4**A). To enhance degradation stability and reduce immunogenicity, we employed N^1^‐methylpseudouridine‐modified Cas9 mRNA (m1Ψ‐mCas9).^[^ ^50^ ^]^ In the previous study, we demonstrated that the combination of TatD and TatE gRNAs (1:1 molar ratio) exhibited the highest efficacy compared to other single and dual gRNA combinations tested.^[^ ^29^ ^]^ The TatDE dual gRNAs target over 50% of HIV‐1 strains and can effectively induce excision across a broad range of HIV‐1 variants. To further enhance stability and mRNA activity, we incorporated the 2′OMe modification into the gRNAs, a strategy adopted in the FDA‐approved Onpattro formulation for treating liver transthyretin amyloidosis.^[^ ^51^, ^52^ ^]^ Subsequently, combining the modified m1Ψ‐mCas9 with TatDE gRNAs is crucial to formulate LNPs that enable CRISPR‐based HIV‐1 DNA editing. Identifying the ideal molar ratio of gRNA to m1Ψ‐mCas9 in these formulations is a significant challenge in optimizing CRISPR editing efficiency. Determining this ratio is crucial, as improper stoichiometry can significantly impair editing efficacy.^[^ ^53^ ^]^ To determine the optimal gRNA/ m1Ψ‐mCas9 ratio, we synthesized a series of LNPs with a gRNA to m1Ψ‐mCas9 molar ratio from 1:1 to 180: 1 and subsequently treated to the JLat 8.4 and J1.1 cells. After 48 h of treatment, PCR and gel electrophoresis were performed to assess CRISPR efficacy. The upper band (3 kb) corresponds to the proviral DNA amplicon in the gel blot. The lower band (428 bp) represents the CRISPR‐edited DNA amplicon (Figure 4C). Excision efficacy was quantified by ImageJ (v 1.54j) and densitometric analysis (Equation S1, Supporting Information), presented in Figure 4D. The LNP with a gRNA to m1Ψ‐mCas9 molar ratio 45:1 showed the highest CRISPR efficacy in JLat 8.4 and J1.1 cells (Figure S6A,B, Supporting Information). Therefore, the optimal gRNA/m1Ψ‐mCas9 molar ratio of 45:1 was used to formulate LNPs and treated to these T cell lines. The T‐LNP demonstrated a higher excision efficacy than C‐LNP in both the T cell lines (Figure 4E–H) and showed a decrease in excision efficacy in the event of CXCR4 blocking (by AMD070) (Figure 4I,J). However, the excision efficacy of T‐LNPs was significantly lower (≈5%) in the promonocytic U1 cell line compared to the T cell lines (Figure S6C,D, Supporting Information). These results are affirmed by their FLuc protein expression levels (Figure 3C). Moreover, T‐LNP shows a dose‐dependent increase in excision efficacy in JLat8.4 and J1.1 cell lines (Figure S6E, Supporting Information). Given that LNPs are reported to be less immunogenic, multiple dosing may increase the likelihood of additive excision efficacy.^[^ ^54^, ^55^ ^]^ To investigate this, T‐LNPs were treated to T cell lines with single, double, and triple doses, with a 48 h interval between each dose (Figure 4B). As expected, excision efficacy elevated with multiple dosing, reaching approximately 80% after the third dose in the JLat 8.4 and J1.1 cell lines (Figure 4K,L; Figure S6F,G, Supporting Information).

### 2.5 HIV‐1 DNA Excision in Infected Lymphoblasts

**Figure 5:**
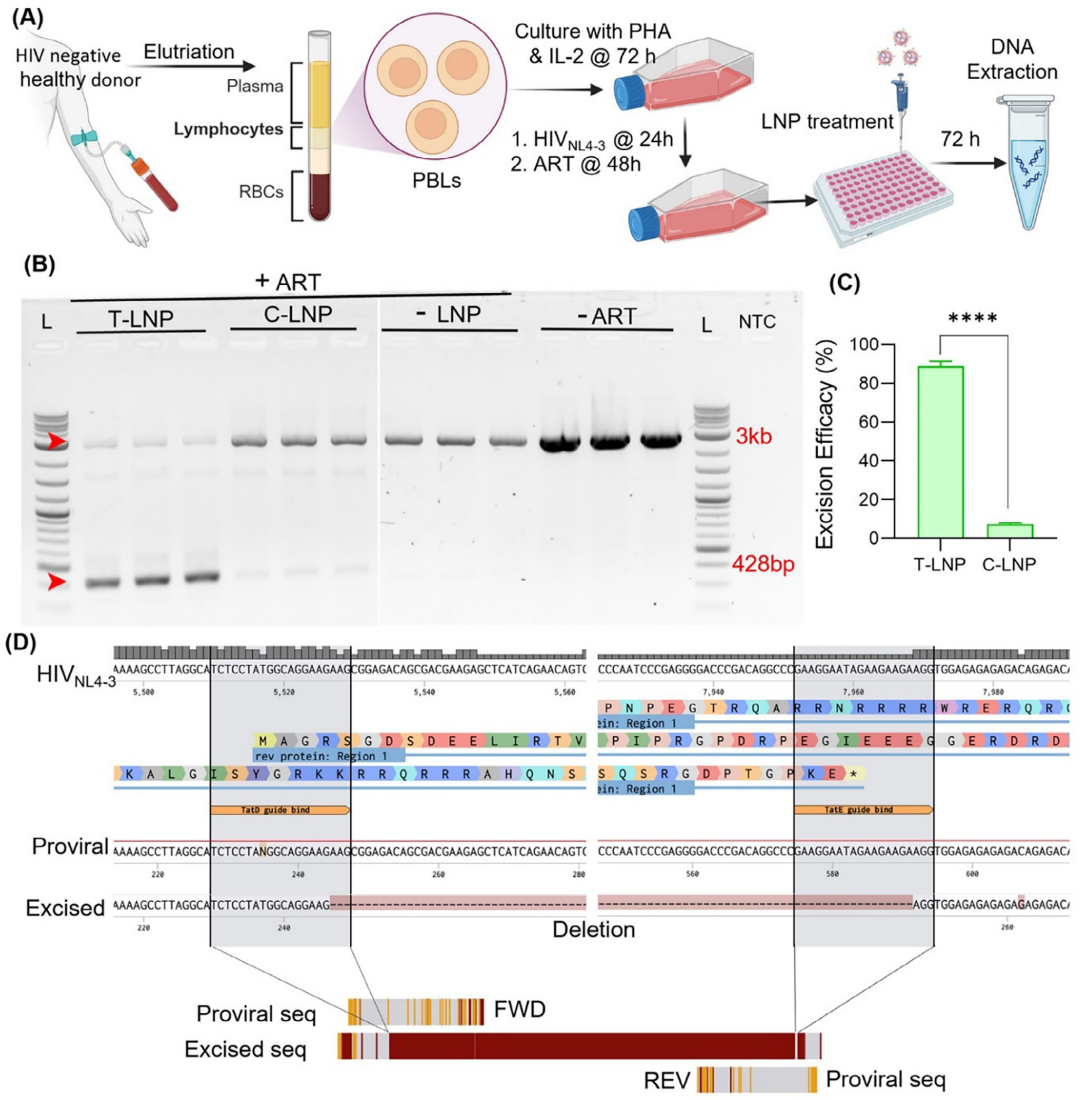
HIV‐1 proviral DNA excision efficacy in lymphoblasts. A) Timeline for elutriation, cell separation, culture, HIV‐1 infection, ART and LNP treatment, and DNA extraction of PBLs collected from healthy, HIV‐1 seronegative donors. Created with BioRender. IL‐2 and phytohemagglutinin‐treated PBLs (lymphoblasts) were infected with HIV‐1_NL4‐3_ at 0.001 MOI for 24 h and subsequently subjected to ART for 48 h. After ART treatment, PBLs were treated with LNPs at an mRNA dose of 8 µg/million cells. PBLs were harvested 72 h after treatment for DNA extraction and PCR. B) PCR gel electrophoresis image shows near‐complete excision of HIV‐1 proviral DNA using T‐LNP. The proviral (3 kb) and excised (428 bp) DNA amplicons are highlighted by red arrowheads. C) The excision efficacy of T‐LNP was quantified using ImageJ and densitometric analysis. D) Excised amplicons were sequenced and confirmed as CRISPR edits by Sanger sequencing. Multiple sequence alignment was performed to align the amplicon sequences with the HIV‐1_NL4‐3_ reference genome (FWD = forward strand, REV = reverse strand). Data are presented as means ± SD (*n* = 3). ^****^ *p* < 0.0001.

The CD4+ T cells serve as essential targets and reservoirs for HIV‐1; however, transfection of these cells poses challenges.^[^ ^56^, ^57^ ^]^ To overcome these challenges, we developed T‐cell‐selective CXCR4‐receptor targeted T‐LNPs to efficiently deliver CRISPR‐m1Ψ‐mCas9/gRNA into these cells. This strategic approach aimed to facilitate efficient uptake of LNPs and subsequent HIV‐1 DNA excision within difficult‐to‐transfect T cells. To examine this notion, human peripheral blood lymphocytes were isolated from HIV‐1 seronegative donors and activated by phytohemagglutinin and interleukin‐2 (PHA/IL‐2) to transform them into lymphoblasts. Lymphoblasts were infected with 0.001 multiplicity of infection (MOI) of HIV‐1_NL4‐3_, an X4 tropic strain (**Figure** **5**A). After 24 h of infection, the cells were treated with a clinically relevant combination of antiretroviral drugs (dolutegravir (DTG), emtricitabine (FTC), and tenofovir alafenamide (TAF)), selected for their ability to inhibit viral replication at various stages of the HIV life cycle.^[^ ^58^ ^]^ Following 48 h of drug exposure, LNPs (8 µg/million cells) were combined with human plasma to simulate physiological conditions in the human body and then incubated with lymphoblasts. After 72 h of treatment, HIV‐infected lymphoblasts were harvested, and DNA was extracted using the PureGene kit to maintain its integrity for downstream analysis. Semi‐nested PCR and gel electrophoresis were utilized to quantitatively assess the HIV‐1 DNA excision efficacy. The results indicated significantly lower proviral DNA in lymphoblasts treated with antiretroviral drugs compared to untreated groups, demonstrating effective suppression of viral replication within the experimental timeframe (Figure 5B). Notably, T‐LNP‐treated cells exhibited nearly 90% excision of HIV‐1 DNA. In contrast, C‐LNPs displayed only a marginal excision of HIV‐1 DNA (5%, Figure 5C). This highlights the impact of CXCR4‐targeted delivery on improving CRISPR‐mediated viral elimination. Furthermore, to confirm the integrity and specificity of CRISPR edits, Sanger sequencing was conducted on both the proviral amplicon (at 3 kb) and excised amplicon (at 428 bp). Sequence alignment against the HIV‐1_NL4‐3_ reference sequence (GenBank accession number AF324493) validated precise CRISPR edits (Figure 5D).^[^ ^59^ ^]^ Overall, this comprehensive study underscores the potential of T‐LNPs to effectively target and eliminate proviral DNA from latently infected and ART‐treated lymphoblasts.

### 2.6 HIV‐1 DNA Excision in Infected ART‐Treated hu Mice

**Figure 6:**
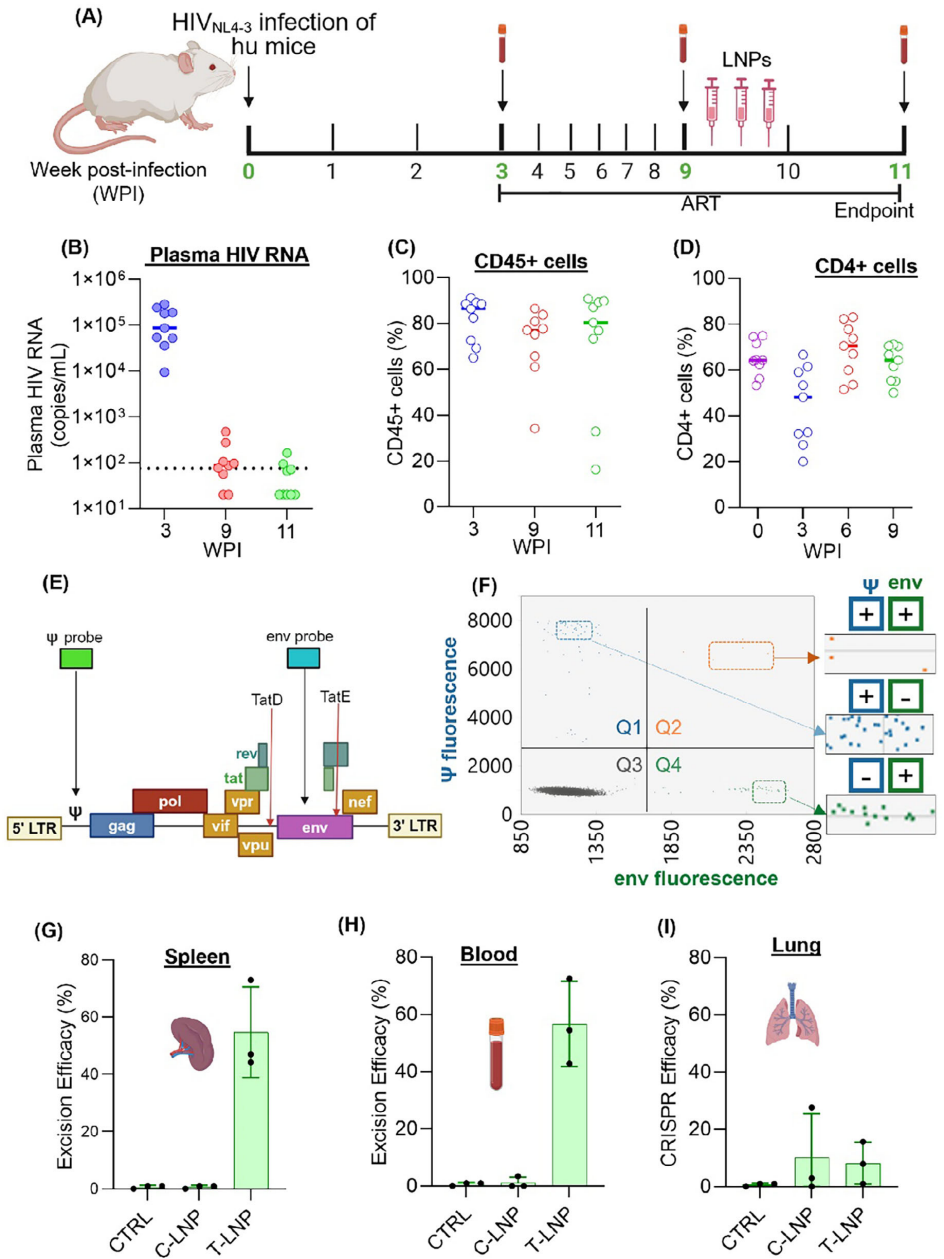
Excision of HIV‐1 proviral DNA in hu mice. A) The study timeline includes blood collection for immune cell profiling, HIV infection, ART and LNP treatments; the study endpoint involves the sacrifice of hu mice (*n* = 3) and the blood and tissue collection. B) The plasma HIV‐1 RNA copies at 3‐, 9‐ and 11‐weeks post‐infection (WPI). C,D) The levels of CD45+ and CD4+ cells in blood were analyzed by flow cytometry. E) The schematic representation of the HIV‐1_NL4‐3_ genome highlights the Ψ and *env* probes together with TatDE binding sites. F) The representative illustration of intact proviral DNA assay (IPDA) 2D duplexed droplet digital PCR plot. Q1(blue), Q2(orange), Q3 (gray) and Q4 (green) quadrants represent Ψ‐single‐positive, Ψ and *env* double‐positive, double‐negative, and *env* single‐positive events, respectively. The HIV‐1 proviral DNA excision efficacy was analyzed by comparing the number of Ψ‐positive and *env*‐positive events. G–I) The HIV‐1 DNA excision efficacy of LNPs in spleen, blood, and lung, analyzed by IPDA assay. T‐LNPs achieved ≈60% HIV DNA excision efficacy in the spleen and blood, while C‐LNPs showed ≤5% excision efficacy. Comparable efficacy was observed in the lungs, but lower than the spleen and blood. Data are presented as means ± SD (*n* = 3). Figures A and E were created with BioRender.

The viral excision efficacy of LNPs was evaluated in HIV‐1 infected and ART‐treated hu mice, which serve as a model with multiple anatomical reservoirs for the virus, including the spleen, lungs, and blood.^[^ ^43^ ^]^ The hu mice were infected with HIV‐1_NL4‐3_ at a dose of 1.5×10⁴ tissue culture infective dose 50 (TCID₅₀) (**Figure** **6**A). At three weeks post‐infection (WPI), plasma viral load measurements revealed an average of 1×10⁵ copies mL^−1^, confirming robust viral replication (Figure 6B). Following this, the animals were given an oral combination ART, which included DTG, FTC, and TAF with DietGel. By 9 WPI (6 weeks post‐ART initiation), we observed a decline in plasma viral load to near‐undetectable levels. This prompted us to randomly divide the mice into T‐LNP, C‐LNP, and LNP untreated control groups. Each LNP group received three intravenous injections (2 mg kg^−1^) at 2‐day intervals (Figure 6A). Animals received continuous oral ART throughout the experiment to suppress viral replication and limit the latent reservoir size.^[^ ^60^, ^61^ ^]^ At 11 WPI, the animals were euthanized, and major organs, along with blood samples, were collected. Portions of the fresh organ tissues were processed for DNA extraction, while the remainder was fixed and subjected to H&E staining. Throughout the study, peripheral human cell populations in mice were monitored via flow cytometry (Figure 6C,D). No significant changes were observed in human CD45⁺ cell levels, although CD4⁺ T cells temporarily declined from 0 to 3 WPI due to HIV‐1 infection. However, ART treatment restored CD4⁺ T cells to their initial levels by 6 WPI, and these levels remained stable until the experimental endpoint (Figure 6D). To preserve minimally sheared DNA for accurate CRISPR editing analysis, we used the PureGene DNA extraction kit (Qiagen) to obtain high‐quality DNA.^[^ ^62^ ^]^ The extracted DNA achieved an average shearing index of 0.32, indicating minimal fragmentation. Semi‐nested PCR was performed on DNA isolated from spleen tissue, and the amplicons were visualized by gel electrophoresis (Figure S7A, Supporting Information). An excised band appeared in two of three T‐LNP‐treated mice and one of three C‐LNP‐treated mice, as confirmed by Sanger sequencing (Figure S7B, Supporting Information). However, HIV‐1 proviral DNA band was not detected in any animal group. This reflected the low abundance of HIV‐1 DNA copies in these infected and ART‐treated mice, which made the PCR ineffective for detection. Additionally, this PCR targets short genomic regions, which cannot clearly distinguish between defective and intact HIV genomes.^[^ ^63^ ^]^ We employed the intact proviral DNA assay (IPDA) to address these limitations. This is a more sensitive method which enables precise differentiation between intact HIV DNA, defective proviruses, and CRISPR‐edited DNA.^[^ ^64^ ^]^ Therefore, this method can provide a reliable assessment of CRISPR‐mediated genome editing efficacy. The schematic illustration of the HIV genome, showing the sites of IPDA probe binding, and a representative IPDA 2D duplex droplet digital PCR plot with four quadrants is presented in Figure 6E,F, respectively. The IPDA demonstrated that T‐LNPs achieved ≈60% CRISPR excision efficacy in the spleen and blood, whereas C‐LNPs displayed much lower efficacy (≤5%) (Figure 6G,H). Comparable efficacy between C‐LNPs and T‐LNPs was also observed in the lungs; however, the efficacy was still lower compared to the spleen and blood (Figure 6I). The higher CRISPR efficacy in the spleen was anticipated, as T‐LNPs were explicitly designed for spleen‐targeted delivery.

Additionally, enhanced efficacy in the blood can be attributed to the CXCR4‐targeted delivery of T‐LNPs to HIV‐infected CD4⁺ T cells. In addition, the nonspecific accumulation of both the LNPs in the lungs resulted in a comparable but lower efficacy relative to that observed in the spleen and blood. It is worth mentioning that the DNA extracted from liver tissue exhibited a high shearing index, likely due to the elevated DNase concentration, which hindered the determination of excision efficacy across all studied groups.^[^ ^65^ ^]^ Overall, the study demonstrated T‐LNP‐mediated targeted proviral excision in blood, spleen, and lungs of HIV‐infected and ART‐treated hu mice.

### 2.7 Toxicology

**Figure 7:**
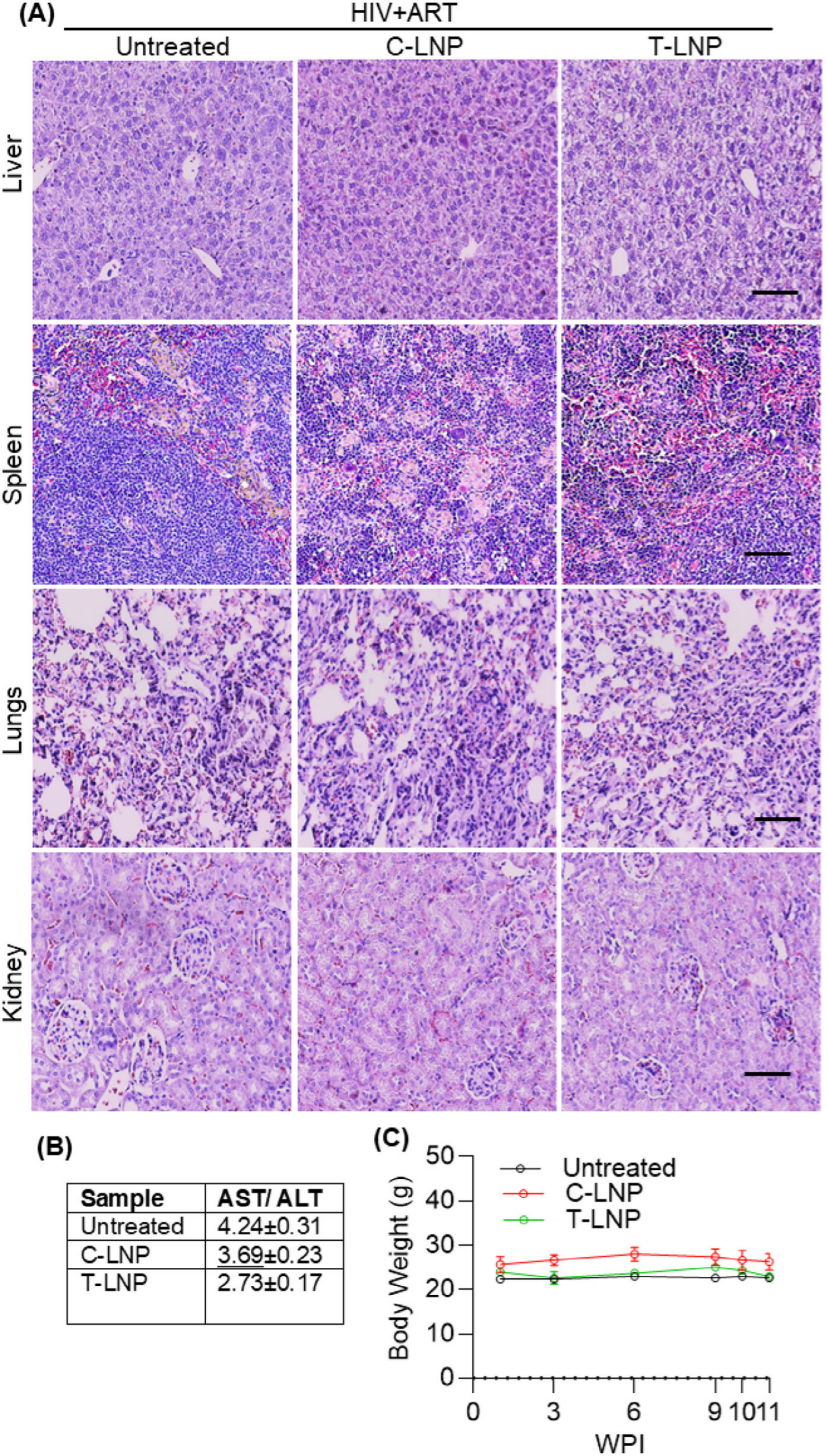
Toxicology evaluation. A) Hematoxylin and eosin staining of liver, spleen, lungs, and kidney tissues from each treatment group imaged at 20× magnification. B) The AST and ALT ratios along the various treatment groups. C) Mice body weight over the treatment period, presented by weeks after infection. No significant changes in body weight were observed, indicating the safety of LNP treatment. Data are presented as means ± SD (*n* = 3).

The toxicological profiles of these LNPs in hu mice were assessed in significant organs, including the lungs, liver, kidneys, and spleen, through histological analysis of hematoxylin and eosin (H&E) stained tissue sections. No histological changes were observed in any of the organs when compared to the untreated control group (**Figure** **7**A). Since C‐LNPs showed liver‐selective mRNA translation and T‐LNPs target the spleen, the functionality of these organs was further evaluated by analyzing hematological parameters. In the LNP‐treated groups, alanine transaminase and alkaline phosphatase levels were lower than those in the untreated control groups, suggesting a potential reduction in liver stress or damage (Table S2, Supporting Information).^[^ ^66^ ^]^ Moreover, albumin, alkaline phosphatase, and total bilirubin showed no significant differences between the groups (Tables S1,S2, Supporting Information). A progressive decrease in AST/ALT values was observed compared to the untreated and C‐LNP‐treated groups. The lowest values were recorded in T‐LNP‐treated mice (Figure 7B). This trend suggests that, although HIV‐1 infection may induce hepatic metabolic activities, treatment with LNPs, particularly T‐LNP, was associated with an improved hepatic enzyme profile (Figure 7B). These findings indicate that the use of MC3 in our T‐LNP formulation did not exacerbate hepatotoxicity in this model; rather, it appeared to mitigate it compared to the untreated group. This effect may be attributed to the splenic tropism of T‐LNPs, in contrast to conventional MC3‐based LNPs, which exhibit liver tropism. The LNP‐treated groups also exhibited no significant changes in neutrophils, lymphocytes, platelet counts, or red blood cell indices, suggesting that spleen function was not compromised (Table S1, Supporting Information). Moreover, no significant changes in body weight were observed between the LNP‐treated and untreated control groups (Figure 7C), further supporting minimal LNP‐related toxicity.

## 3 Conclusion

We successfully synthesized CXCR4 ligand‐conjugated lymphoid tissue‐targeting LNPs. These LNP exhibited higher luminescence in the spleen than in liver tissue, confirming splenic tissue‐targeted mRNA translation. The LNP revealed CXCR4‐expressing latently infected CD4+ lymphocytic cells targeted enhanced mRNA translation compared to promonocytic cells. With an optimal gRNA/m1Ψ‐mCas9 molar ratio (45:1), targeted LNPs achieved significantly higher HIV‐1 DNA excision efficacy in lymphocytes. Repeated dosing led to a cumulative increase in excision efficacy of ≈80%, addressing the limitations of the current AAV‐based delivery system. In the infected hu mice, the sequential treatment of ART and targeted LNPs resulted in ≈60% HIV‐1 DNA excision efficacy in blood and splenic tissue. Notably, the toxicological evaluations confirmed their safety profile, supporting their feasibility for HIV‐1 therapy. Collectively, these findings highlight LNP formulations as a robust and promising platform for targeted CRISPR‐gRNA/m1Ψ‐mCas9 delivery to HIV‐1 reservoirs with excision of latent proviral DNA. This study represents a significant step toward achieving a functional HIV‐1 cure. Furthermore, the CRISPR‐gRNA/m1Ψ‐mCas9 delivery strategy used here can also be applied to treat other life‐threatening diseases.

## 4 Experimental Section

### Synthesis of CXCR4 Peptide Conjugated DSPE‐PEG

The cyclic peptide, cyclo(Nal‐Gly‐(D‐Tyr)‐Orn‐Arg‐), was chosen as the CXCR4‐targeting ligand. To synthesize the cyclic peptide conjugated to DSPE‐PEG, the amine group (in the ornithine) of the peptide was reacted with the activated carboxyl group of DSPE‐PEG (2000) carboxy NHS. In brief, the cyclic peptide (2.86 mg, 4.16 µmol) was dissolved in 100 µL of anhydrous DMSO with DIPA (5 µL) in a Schlenk tube and stirred at room temperature for 30 min. After that, DSPE‐PEG (2000) carboxy NHS (10 mg, 3.47 µmol) was added to the reaction mixture and stirred overnight at room temperature. The progress of the reaction was monitored by TLC using a mixture of MeOH/DCM (1:5 by volume) as the eluent. Upon completion, the reaction mixture was diluted with DI water, placed in a Float‐A‐Lyzer (cellulose acetate, 0.5–1 kDa cutoff), and dialyzed against DI water for 2 days. The dialyzed solution was then lyophilized to obtain a white solid, which was characterized by ^1^H NMR and mass spectrometry.

### Formulation of LNPs

LNPs were prepared by mixing three volumes of mRNA in acetate buffer (pH 4.5) with one volume of lipid solution in ethanol using a microfluidic mixer (Precision Nanosystems Inc.). The mRNA concentration in acetate buffer was maintained at 200 µg mL^−1^. For control LNPs, the lipid solution consisted of D‐Lin‐MC3‐DMA (50 mol%), DSPC (10 mol%), β‐Sitosterol (37 mol%), and DMG‐PEG 2000 (3 mol%). For targeted LNPs, the lipid solution included D‐Lin‐MC3‐DMA (50 mol%), DSPC (10 mol%), β‐Sitosterol (37 mol%), DMG‐PEG 2000 (2.8 mol%), DSPE‐PEG‐CycPep (0.2 mol%), and DOPS (5 mol% of total lipids). For all formulations, the N/P ratio was maintained at 4. Following LNP formation, the particles were dialyzed against PBS at 4 °C for 18 h using a Float‐A‐Lyzer (cellulose acetate, 3.5–5 kDa cutoff). Subsequently, the LNPs were concentrated with an Amicon centrifugal filter (MWCO 10 kDa) at 2000 ×g for 15 min. To prepare the Cy5.5 dye‐labeled LNPs, DSPE‐PEG_2000_‐N‐Cy5.5 was added to the lipid solution, and the aforementioned procedure was followed.

### Size and Zeta Potential (ζ‐Potential)

To measure the size of the LNPs, the samples were diluted 100‐fold with PBS. The diluted solutions were transferred into polystyrene cuvettes and analyzed for size and ζ‐potential using dynamic light scattering (DLS) with a Malvern Zetasizer Nano ZS instrument.

### LNP Plasma Stability

The stability of the LNPs in plasma was assessed by incubating them with 10% plasma at 37 °C. Their size and concentration variations were monitored using nanoparticle tracking analysis (Malvern NanoSight NS300 instrument).

### Cryo‐TEM Analysis

The carbon‐coated TEM grid was glow‐discharged to enhance its surface energy. It was then transferred to the FEI Vitrobot Mark III sample chamber, which was set to maintain a 100% humidified environment at 26 °C. A 2 µL aliquot of PBS‐diluted LNPs was applied to the grid, which was subsequently plunged into liquid ethane to vitrify the PBS solution. The vitrified grid was imaged using an FEI Tecnai Spirit BioTWIN transmission electron microscope operating at 120 kV. During imaging, the sample temperature was maintained between −180 and −175 °C.

### TNS Assay

The hydrophobic fluorescent dye TNS was used to determine the pKa of the LNPs. Initially, different buffer solutions with pH values ranging from 2 to 10 were prepared. Buffers with pH 2–5.5 were prepared using 20 mm citric acid/NaOH and 150 mm NaCl. Buffers with pH 6–8 were prepared using 20 mm sodium dihydrogen phosphate/NaOH and 150 mm NaCl, while buffers with pH 8.5–10 were prepared using 20 mm Tris/HCl and 150 mm NaCl. In a 96‐well plate, 10 µL of LNP and 5 µL of TNS solutions (0.6 mm) were incubated with 185 µL of the respective buffer solutions in triplicate. The plate was then wrapped in aluminum foil and placed on a shaker at 250 rpm for 15 min. Fluorescence intensity (Ex/Em = 321/447 nm) was recorded using a benchtop plate reader (Molecular Devices, SpectraMax M3) and plotted against pH. The pKa was defined as the pH at which the LNP exhibited half of the maximum fluorescence intensity.

### mRNA Encapsulation Efficiency

Encapsulation efficiency was measured using the RiboGreen assay. Briefly, 10 µL of LNP was diluted with 1× TE buffer to a final volume of 250 µL. In a 96‐well plate, 50 µL of the diluted LNP was separately incubated with an equal 1×TE buffer and 1× Triton. The plate was then incubated at 37 °C for 10 min. Subsequently, 100 µL of 1% (v/v) RiboGreen reagent (Thermo Fisher) was added to each well in the dark. Fluorescence intensity was measured using a benchtop plate reader at an excitation/emission wavelength of 485/528 nm. The amount of encapsulated mRNA in the LNP was quantified using a standard curve generated from known concentrations of the same mRNA.

### Flow Cytometry Analyses

The CXCR4 receptor population was determined by flow cytometry using an anti‐human CXCR4 antibody. U1, JLat 8.4, and J1.1 cell lines were seeded in flow tubes at a density of 1×10⁶ cells mL^−1^. The cells were centrifuged at 650 ×g for 5 min and resuspended in LIVE/DEAD reagent (1:1000 dilution; Thermo Fisher Scientific, L23105) for 30 min. After incubation, the cells were centrifuged, washed with PBS, and resuspended in flow cytometry staining buffer containing anti‐human CXCR4 antibody (1:200 dilution; Bio‐Techne, Human CXCR4 Alexa Fluor 488‐conjugated Antibody) for another 30 min. The cells were washed three times with PBS, resuspended in the flow cytometry fixation buffer, and analyzed using FlowJo (v10.10, BD Biosciences).

### Cell Culture

Latently HIV‐1‐infected U937 pro‐monocytic cells (NIH ARP #165), human T lymphocyte JLat 8.4 full‐length cells (NIH ARP #9847), and HIV‐1 lymphadenopathy‐associated virus (LAV)‐infected J1.1 cells (NIH ARP #1340) were cultured in RPMI‐1640 medium supplemented with 10% fetal bovine serum and 1% penicillin‐streptomycin in vented culture flasks at 37 °C with 5% CO₂. For subsequent experiments, each cell line was plated at a density of 0.1×10^6^ cells per well in 96‐well plates.

Human peripheral blood lymphocytes (PBLs) were obtained from HIV‐1 and hepatitis‐B seronegative donors via leukapheresis followed by countercurrent centrifugal elutriation. PBLs were seeded at 1.5 ×10^6^ cells mL^−1^ and cultured for 72 h in RPMI‐1640 medium supplemented with human interleukin‐2 (IL‐2, 100 IU/mL; PeproTech, 200–02) and 1.5% (v/v) phytohemagglutinin M (PHA‐M, Gibco, 10 576 015). After that, PBLs were maintained in IL‐2‐supplied RPMI‐1640 medium.

### Measures of Cell Viability

Cells were seeded at a density of 1×10^5^ cells per well in a 96‐well plate and treated with a serial dilution of LNP corresponding to mRNA doses ranging from 2 to 0.15 µg/million cells. Forty‐eight hours after LNP treatment, 20 µL of CellTiter‐Blue (CTB, Promega) reagent was added to each well and incubated for 2 h. Fluorescence intensity (FI) was measured for each well (Ex/Em = 560/590 nm) using a SpectraMax M3 plate reader (Molecular Devices, CA). Cell viability (%) was calculated using the formula: ([FI]_treated_ × 100) / [FI]_untreated_).

### Cell Uptake Assays

Cellular uptake of Cy5.5‐labeled LNPs was observed using a confocal microscope (LSM 800 Zeiss, Jena, Germany). Cells were seeded in 48‐well plates at a density of 0.1×10^6^ cells per well. To block the CXCR4 receptor, a subset of cells was preincubated with 12 µm AMD070 for 30 min. LNPs containing an equivalent concentration of Cy5.5 dye were then incubated with both AMD070‐treated and untreated cells. After 4 h of incubation, each group of cells was transferred into separate 1.5 mL Eppendorf tubes, centrifuged at 650 ×g for 5 min, and washed with PBS. The cell cytoskeleton and nuclei were stained with Phalloidin‐iFluor 488 (1:1000 dilution; Abcam, ab176753) and DAPI (1:1000 dilution; Thermo Fisher Scientific, D1306), respectively. Excess staining reagents were removed by washing three times with PBS, and the cells were resuspended in PBS. The cell suspensions were then mounted on glass slides, covered with coverslips, and imaged using confocal microscopy.

### Luciferase Assays

The luciferase assay used the Promega luciferase assay system (E1501). In a 96‐well plate, 0.1 × 10^6^ cells were seeded per well and treated with LNPs containing firefly luciferase (FLuc) mRNA. LNPs containing FLuc mRNA were treated at 2–0.065 µg/10^6^ cells and incubated for 48 h at 37 °C in 5% CO_2_ atmosphere. After the incubation period, the cells were centrifuged at 650 × g for 5 min, washed with PBS, and lysed by incubation with 100 µL of 1x lysis buffer (Promega) on a shaking platform for 15 min. Following lysis, 100 µL of the Promega Luciferase assay reagent was added to the cell lysate, and the luminescence was measured according to the manufacturer's protocol. For CXCR4 inhibition, cells were pre‐incubated with AMD070 (12 µm) for 30 min. After the pre‐incubation, the cells were washed with fresh media and treated with LNPs as described in the luciferase assay protocol.

### Evaluation of Tissue Luciferase Expression

LNPs were sterilized using a 0.2 µm PES syringe filter. Sterile LNPs were administered to the mice by tail vein injection at 0.5 mg kg^−1^ FLuc mRNA. Six hours post‐administration, mice were injected intraperitoneally with D‐Luciferin (150 mg kg^−1^). Twelve minutes after injection, the mice were anesthetized, and whole‐body imaging was conducted using an in vivo imaging system (IVIS, PerkinElmer, Waltham, MA). Following imaging, the mice were euthanized, and multiple organs, including the lungs, heart, gut, kidneys, spleen, liver, and lymph nodes, were dissected and imaged under the IVIS.

### PCR Analyses

DNA was extracted from cell and tissue samples using the Puregene Tissue Core Kit (QIAGEN) according to the manufacturer's protocol and quantified using the NanoDrop One (Thermo Fisher Scientific, Waltham, MA, USA). The extracted DNA was then subjected to semi‐nested PCR. In the first step, a reaction mixture was prepared by combining 1 µL of DNA template (100 ng), 10 µL of Invitrogen Platinum SuperFi PCR Master Mix, 1 µL of each forward (P‐449: ACACAAGTAGACCCTGACC TAGCAGAC) and reverse (P‐450: CCCAGAAGTTCCACAATCCTCGTT ACAATC) primer, and 7 µL of nuclease‐free water to achieve a final volume of 20 µL. The reaction mixture was initially heated at 98 °C for 30 s, followed by 15 cycles of 98 °C for 5 s, 60 °C for 30 s, and 72 °C for 90 s, with a final extension at 72 °C for 5 min. The PCR product was then diluted 1:10 with nuclease‐free water. For the second step of nested PCR, 2 µL of the diluted PCR product, 10 µL of Master Mix, 1 µL of each forward (P‐451: CTTGGGCAGGAGTGGAAGCCATAATAAG) and reverse (P‐450) primer, and 6 µL of nuclease‐free water were mixed to make a final volume of 20 µL. The reaction was subjected to 35 cycles using the same conditions as in the first step. The gel electrophoresis (1.2% agarose gel, 75 V for 35 min.) was performed on the PCR amplicons and visualized by Invitrogen iBright Imagers. The band intensities of the proviral amplicon (3 kb) and excised amplicon (428 bp) were quantified using ImageJ, and CRISPR efficacy was evaluated by densitometric analysis using Equation (1). The amplicons were then extracted using the NucleoSpin Gel and PCR Clean‐up Kit (MACHEREY‐NAGEL) and sequenced via Sanger sequencing. The CRISPR edits were analyzed using the Synthego Interference of CRISPR Edits Analysis v3 tool.

(1) $$Excision\;Efficiency\;\left(\%\right)=\frac{Iex\times 100}{\left(Iex+Ipvr\right)}$$

I*_ex_* = Band intensity of the excised DNA amplicon, I*_pvr_* = Band intensity of intact proviral DNA amplicon.

### Animal Studies

NSG‐hu mice were used for viral excision therapy.^[^ ^14^ ^]^ Briefly, NSG (NOD.Cg‐Prkd^cscid^ Il2rgt^m1Wjl^/SzJ) mice (animals of random sex, weighing 25–30 g), obtained from Jackson Laboratories (Bar Harbor, ME), were maintained under pathogen‐free conditions. The animal protocols were approved by the Institutional Animal Care and Use Committee of the University of Nebraska Medical Center (UNMC), and all procedures followed the Public Health Service (PHS) Policy on Humane Care and Use of Laboratory Animals by the National Institutes of Health. At 20 weeks of age, a pre‐infection blood draw was conducted to confirm the presence of human CD45+, CD3+, CD19+, CD4+, CD8+, and CD14+ cells by flow cytometry. Mice (>30% CD45+ human cells) were then infected with HIV‐1_NL4‐3_ via intraperitoneal injection at a dose of 1.5×10⁴ TCID50/mouse. Peripheral blood samples were collected via the submandibular vein at 3‐, 9‐, and 11‐weeks post‐infection (WPI) for flow cytometry analysis and viral load measurement. Upon confirming HIV‐1 infection at 3 WPI, the mice were subjected to ART with DietGel food (Clear H_2_0), supplemented with FTC, TAF, and DTG. After viral suppression was confirmed at 9 WPI, the mice were divided into 3 groups: T‐LNP and C‐LNP treatment groups, and ART‐only control (*n* = 3/ group). LNP treatment groups received three subsequent LNP injections via tail vein, every two days at a dose of 2 mg kg^−1^ mRNA. The ART‐supplemented diet was continued for all mice throughout the study, and their body weight was measured weekly. At 11 WPI, mice were euthanized, and peripheral blood and organs were collected for analysis. Complete blood count and immune profiles were assessed using the VetScan HM5 Hematology Analyzer and VS2 Chemistry Analyzer (Zoetis), respectively. The toxicity evaluation was conducted at the end of the experiment, specifically at 11 WPI, following the same dosing regimen of 2 mg kg^−1^ (administered in three doses), as described above.

### Immunohistochemistry

The mouse organs were perfused with PBS, followed by 4% PFA, then fixed overnight using Epredia STP 120 Spin Tissue Processor (Epredia, Thermo Fisher Scientific, Waltham, MA, USA) and embedded in paraffin. Tissue sections (5 µm thick) were cut from the paraffin blocks and mounted on glass slides. Sections were counterstained with Mayer's hematoxylin. Images were acquired using Echo Revolution Microscope (San Diego, CA).

### Intact Proviral DNA Assays

Genomic DNA was extracted from fresh tissues of euthanized mice using Puregene Tissue Core kit (QIAGEN,158 063) following the manufacturer's instructions. DNA concentration was measured using NanoDrop One (Thermo Fisher Scientific, Waltham, MA, USA). Intact and defective copies of HIV‐1 were analyzed by the digital droplet PCR (ddPCR) using the intact proviral DNA assay (IPDA).^[^ ^63^, ^67^ ^]^ Briefly, RPP30 and HIV‐1 reactions were carried out in parallel, and outputs were normalized to the quantity of DNA used for each group. For the ddPCR study, the reaction mix consisted of the DNA template (10 ng for RPP30 and 1000 ng for HIV‐1 reactions), ddPCR Supermix for Probes (no dUTPs, BioRad), primer‐probe assays (Custom TaqMan Gene Expression Assays, Applied Biosystems), and nuclease‐free water. Primer probe sequences used are as follows (5′–3′): RPP30 Forward Primer‐GATTTGGACCTGCGAGCG, Reverse Primer‐ GCGGCTGTCTCCACAAGT, RPP30 Probe sequence‐ VIC‐CTGACCTGAAGGCTCT‐MGBNFQ; RPP30 Shear Forward Primer–CCATTTGCTGCTCCTTGGG, RPP30 Shear Reverse Primer‐ CATGCAAA GGAGGAAGCCG, RPP30 Shear Probe Sequence‐ FAM‐ AAGGAGCAA GGTTCTATTGTAG‐MGBNFQ; HIV‐1 Ψ Forward Primer‐ CAGGACTCGG CTTGCTGAAG, HIV‐1 Ψ Reverse Primer‐ GCACCCATCTCTCTCCTTCTAGC, HIV‐1 Ψ Probe‐ FAM‐ TTTTGGCGTACTCACCAGT‐ MGBNFQ; HIV‐1 *env* Forward Primer‐ AGTGGTGCAGAGAGAAAAAAGAGC, HIV‐1 *env*  Reverse Primer‐ GTCTGGCC TGTACCGTCAGC, HIV‐1 *env* Probe‐ VIC‐CCTTGGGTTCTTGGGA‐ MGBNFQ. Droplets were prepared using an automated droplet generator (BioRad) and subjected to the following cycling conditions: 95 °C for 10 min; 45 cycles of (94 °C for 30 s, 59 °C for 1 min) and 98 °C for 10 min. Droplets were read on QX200 droplet reader (BioRad) and analyzed using QuantaSoft Analysis Pro software (BioRad, version 1.4). Three technical replicates were performed for each sample and merged before analysis to increase the dynamic range of IPDA. The DNA shearing index (DSI) and cell equivalent number were calculated using the readout from RPP30 reactions. HIV‐1 copies from the HIV‐1 reaction readout were then corrected for shearing and normalized to 10^6^ cells before calculating the CRISPR efficacy. The Ψ probe detects the 692–797 base region, while the *env* probe detects the 7736—7851 base region of the HIV‐1 genome (HXB2 reference strain).^[^ ^68^ ^]^ The detection region of the *env* probe overlaps with the TatDE gRNA binding region (5964–8434 bases). Therefore, after TatDE gRNA‐mediated CRISPR editing, the HIV‐1 DNA loses the *env* probe binding region. The CRISPR efficacy was assessed by comparing the readout of the Ψ and *env* probes, assuming the formation of an equal ratio of 3′ and 5′ defective proviruses. The excision efficacy (%) was calculated from the HIV‐1 reaction's readouts and using Equation (2).

(2) $$Excision\;Efficacy\;\left(\%\right)=\frac{\left(\Psi -env\right)\times 100}{\Psi }$$

Ψ = Ψ positive, *env* negative events and *env* = *env* positive, Ψ negative events.

### Statistical Analyses

Statistical analysis was conducted using Prism 10 (GraphPad Prism 10.2.2.397, GraphPad Software, Inc., San Diego, CA) with data from at least three independent experiments, as indicated. Results are shown as mean ± standard deviation. An unpaired, one‐tailed Student's *t*‐test was used to evaluate the statistical difference between the two groups. A p‐value of less than 0.05 was deemed statistically significant. Statistical significance was determined using either an unpaired Student's *t*‐test or one‐way ANOVA. Significance levels were defined as ^*^ *p* ≤ 0.05, ^**^ *p* ≤ 0.01, ^***^ *p* ≤ 0.001, and ^****^ *p* ≤ 0.0001.

## Conflict of Interest

The authors declare that Dr. Howard Gendelman is co‐founder of Exavir Therapeutics, Inc. The biotechnology company is developing ultra‐long‐acting drugs. The drugs in development are not linked to those created in the current report. All other authors declare no competing interests.

## Author Contributions

S.P. and L.A.Z. contributed equally to this work. S.P. and H.E.G. conceptualized the idea and the research study design. S.P. performed the experiments and wrote a first draft of the manuscript. L.A.Z. conducted experiments and contributed to the writing of the manuscript and to the virologic, cell, and animal model experiments. C.Z. and P.D. planned, organized, and co‐executed the animal studies with S.P., HE.G., and L.A.Z. S.G. prepared the antiretroviral drugs for treatments and testing of the hu mice for biodistribution studies. M.P. performed the virological experiments with L.A.Z. H.E.G. edited the manuscript. All authors have read and approved the final version of the manuscript.

## Supporting information



Supporting Information

## Data Availability

The data supporting the findings of this study are available in the article and its supplementary Information.
