# Supplementary material for: Lymphoid and CXCR4 Cell Targeted Lipid Nanoparticles Facilitate HIV‐1 Proviral DNA Excision
Source: Adv Healthc Mater. 2025 Jul 14;14(25):2501190. doi: 10.1002/adhm.202501190 (PMC12477575; doi:10.1002/adhm.202501190)
Supplement: Supplementary file 1 — Supporting Information [file ADHM-14-0-s001.docx]

**Supplementary Information for “Lymphoid and CXCR4 Cell Targeted Lipid Nanoparticles Facilitate HIV-1 Proviral DNA Excision”**

Sudipta Panja^1#*^, Lubaba A. Zaman^1#^, Chen Zhang^1^, Milankumar Patel^1^, Santhi Gorantla^1^, Prasanta K. Dash^1^ and Howard E. Gendelman^1*^

^1^Department of Pharmacology and Experimental Neuroscience, University of Nebraska Medical Center, Omaha, NE, 68198, USA.

^#^Authors contributed equally to this work.

*Correspondence

For Review and Co-Correspondence: Howard E. Gendelman, M.D. Department of Pharmacology and Experimental Neuroscience, University of Nebraska Medical Center, Omaha, NE 68198; Phone 402-559-8920; Fax 402-559-3744; Email [hegendel@unmc.edu](mailto:hegendel@unmc.edu)

For Co-Correspondence: Sudipta Panja, PhD. Department of Pharmacology and Experimental Neuroscience, University of Nebraska Medical Center, Omaha, NE 68198; Phone 512-576-2995; Email [sudipta.panja@unmc.edu](mailto:sudipta.panja@unmc.edu)

This file includes Supplementary figures S1 to S7, Tables S1 and S2


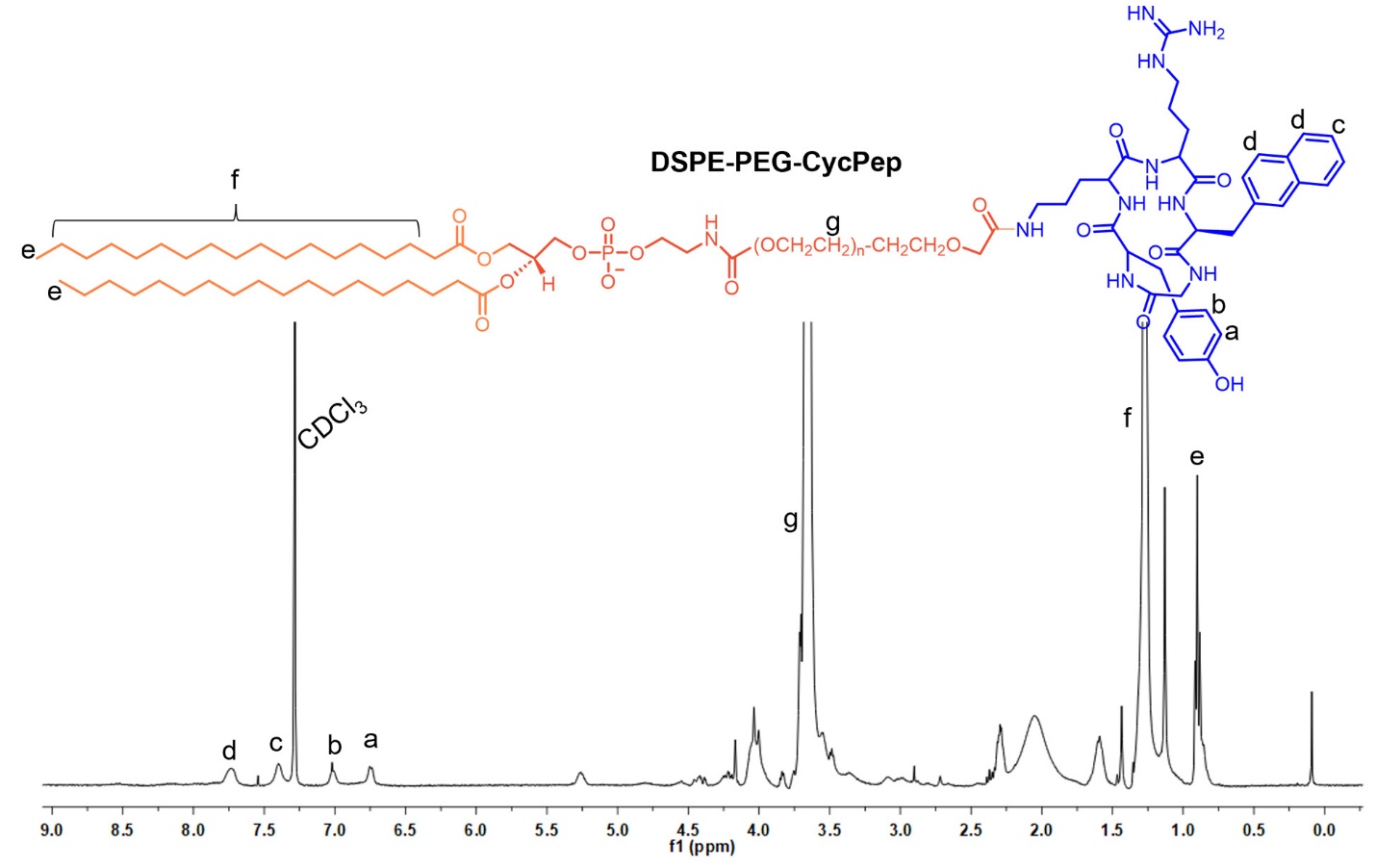
**Figure S1.** ^1^H NMR spectrum of DSPE-PEG-CycPep obtained by dissolving in CDCl₃.


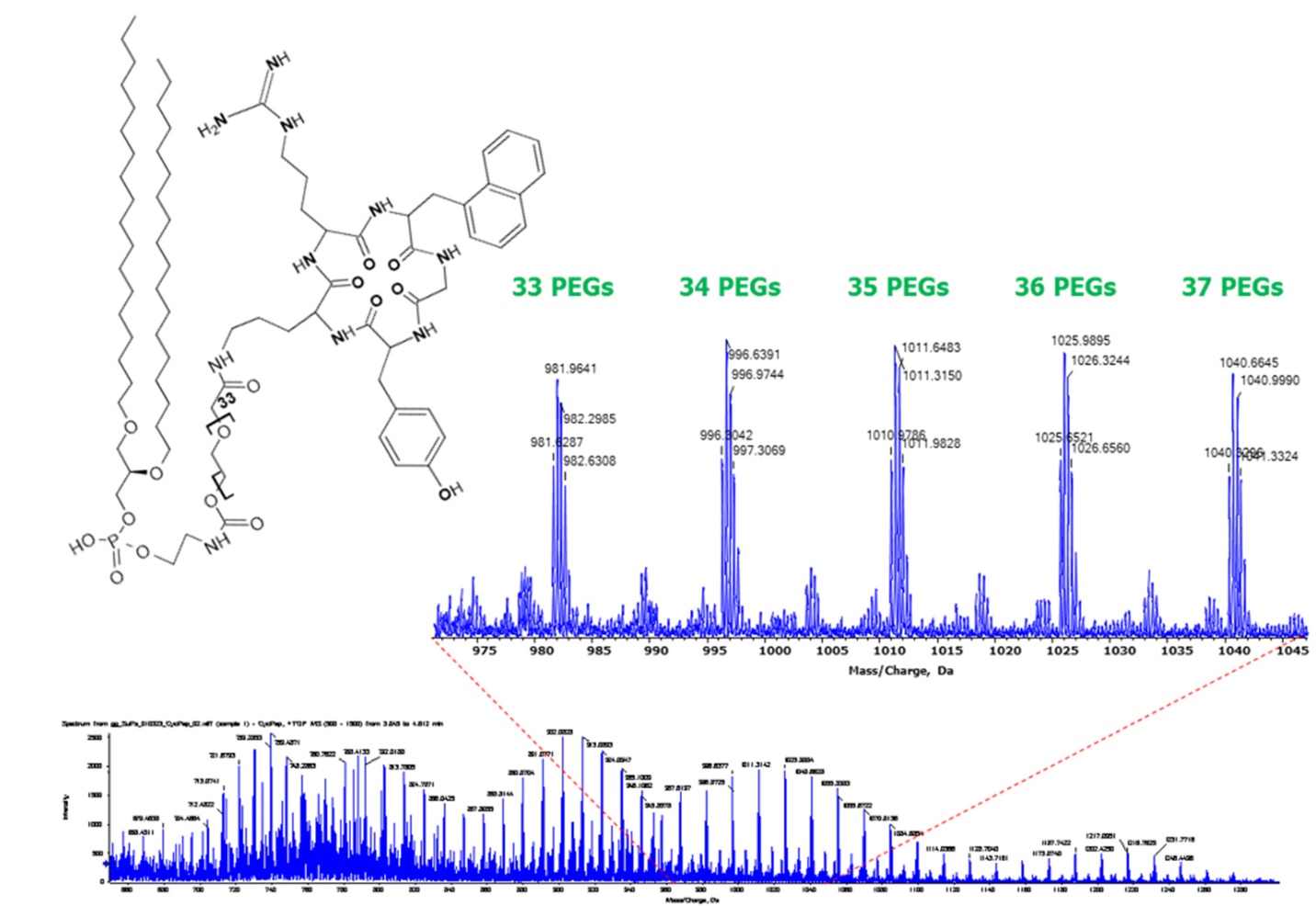


# **Figure S2.** HRMS spectrum of DSPE-PEG-CycPep, with a zoomed-in view showing the [M+H]^3+^ product.


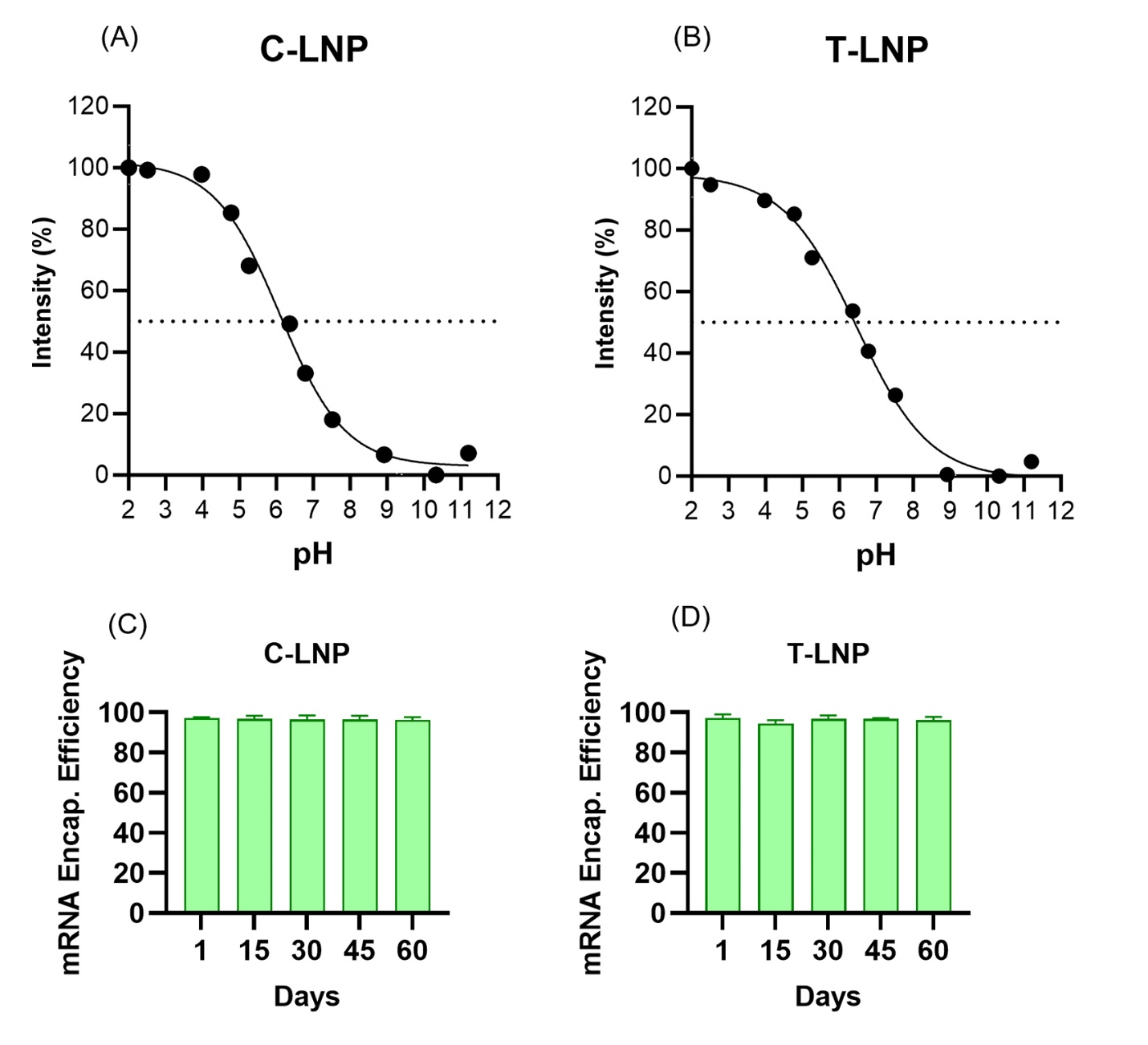


**Figure S3.** (A, B) The mRNA encapsulation efficiency of C-LNP and T-LNP was examined over 60 days, and no significant change was observed in encapsulation efficiency. (C, D) Apparent pKa values of LNPs were determined using the TNS assay, revealing a pKa range between 6.0 and 6.4. Data are presented as means ± SD (n = 3).

**Figure S4.** (A) Quantification of luminescence intensity in the lungs, liver, and spleen of T-LNP-treated hu mice. (B, C) The mRNA translation efficiency of C-LNP containing either DSPE-PEG-CycPep or DOPS in humanized mice. Data are presented as means ± SD (n = 3).


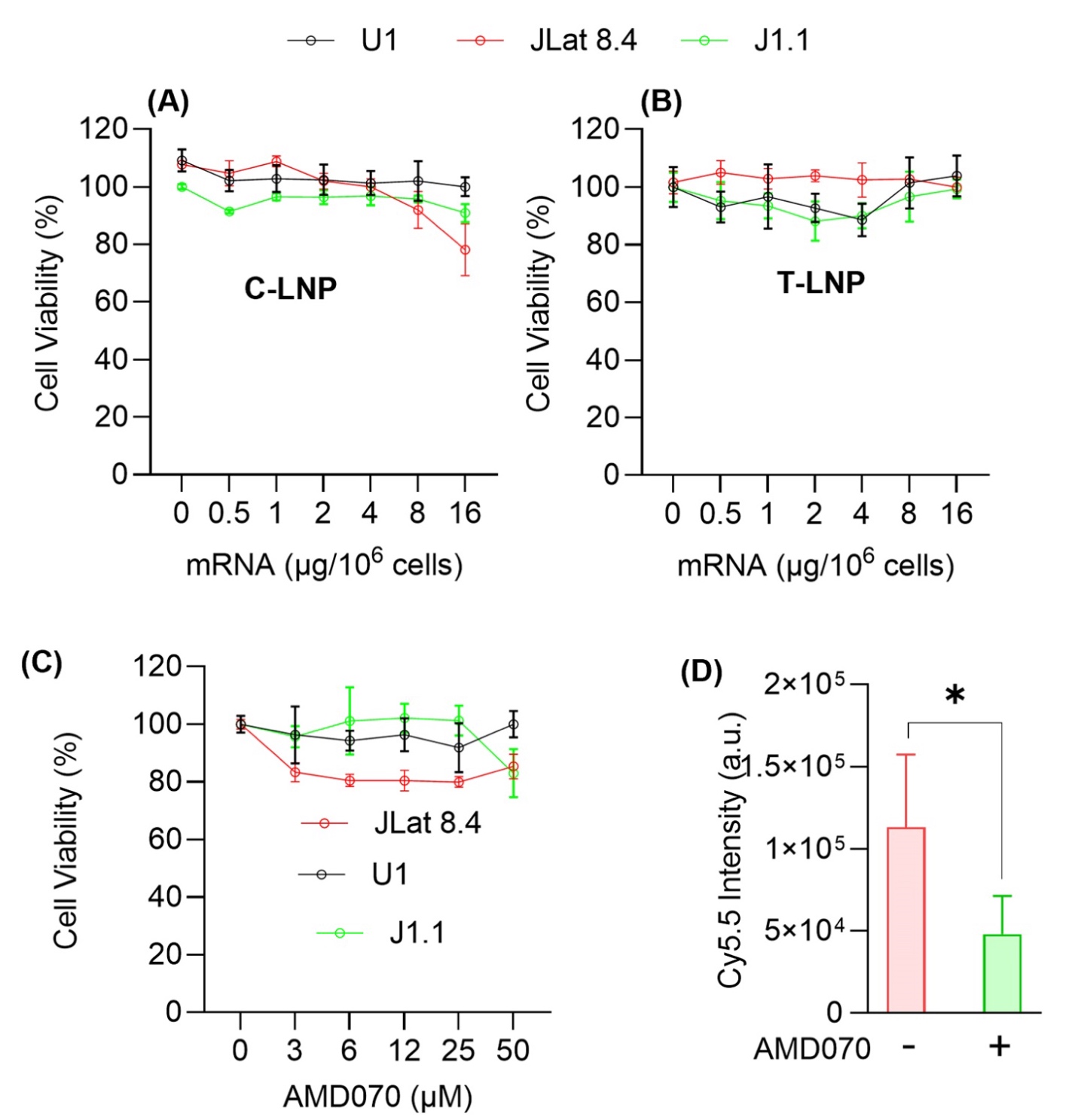


**Figure S5.** (A, B) The dose-dependent cell viability of LNPs across different cell lines. Cells were treated with LNPs, and cell viability was assessed 48 h post-treatment using the CellTiter-Blue™ assay. (C) Quantification of Cy5.5 signal intensity from fluorescence microscopy images of AMD070 treated and untreated J1.1 cells. Data are presented as means ± SD (n = 3). *p < 0.05


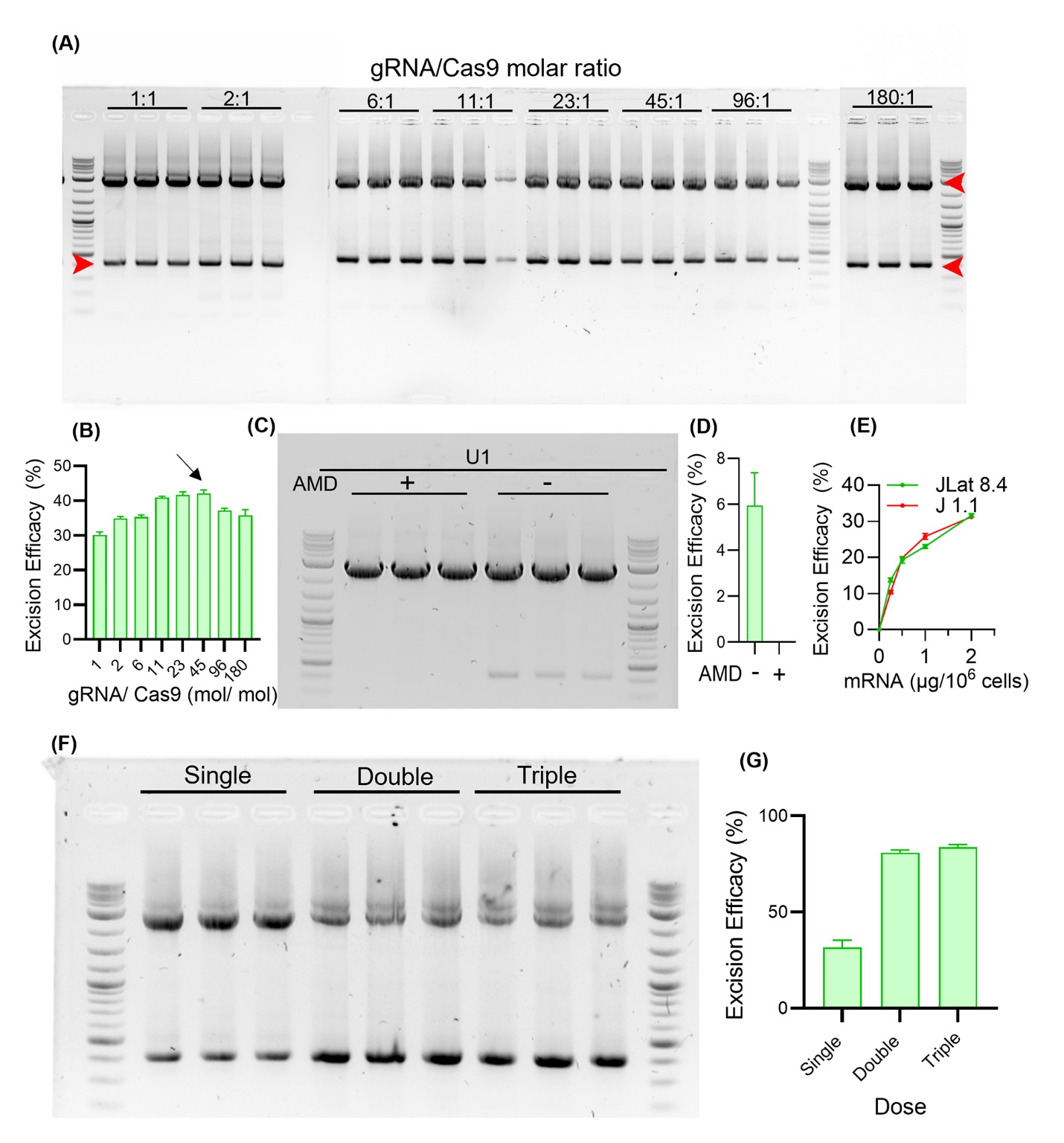


**Figure S6.** (A) PCR gel electrophoresis image of DNA extracted from lymphocytic J1.1 cells treated with LNPs (1µg/10⁶ cells) formulated with varying gRNA/Cas9 mRNA mole ratios. Red arrowheads denote the proviral DNA amplicon (3 kb) and the excised DNA amplicon (428 bp). (B) Quantifying HIV-1 DNA excision efficiency of LNPs formulated with different gRNA/Cas9 mRNA mole ratios, analyzed using ImageJ and densitometric analysis. (C, D) Comparison and quantification of DNA excision efficacy to assess the effect of AMD070 pretreatment on the HIV-1 DNA excision efficiency of T-LNPs in the U1 cell line. (E) The dose-dependent increase in excision efficacy of T-LNP in JLat 8.4 and J1.1 cell lines. (F, G) PCR gel electrophoresis image and quantification of HIV-1 DNA excision efficacy following multiple doses of LNP treatment in the J1.1 cell line. Data are presented as means ± SD (n = 3).

**
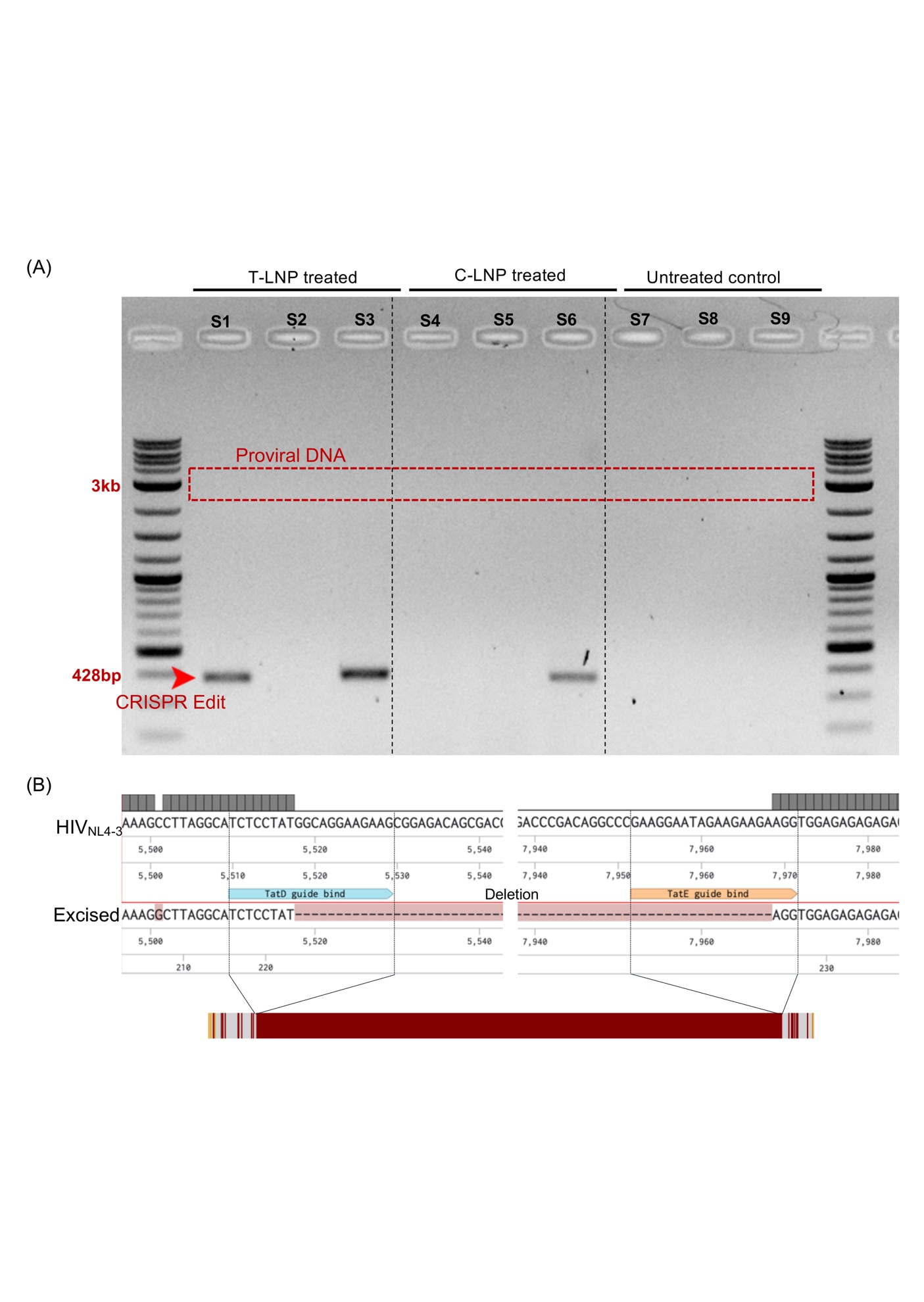
**

**Figure S7.** (A) PCR gel electrophoresis image of DNA extracted from splenic tissue of HIV-1-infected and ART-treated hu mice. (B) Excised amplicons were sequenced and confirmed by Sanger sequencing. The red dotted line box denotes the position of the posited intact proviral DNA amplicon (3kb). The red arrowhead denotes the excised DNA amplicons (428 bp).

**Table S1.** Complete blood count analysis of HIV-1 infected, ART, and LNP treated hu mice (n = 3).

|  | **HIV-1 infected and ART-treated** | | |
| --- | --- | --- | --- |
|  | **Untreated** | **C-LNP** | **T-LNP** |
| **WBC** (10^9^/L) | 5.48 ± 1.80 | 5.29 ± 1.49 | 7.33 ± 1.47 |
| **LYM** (10^9^/ L) | 3.70 ± 1.46 | 4.11 ± 1.88 | 4.49 ± 1.17 |
| **MON** (10^9^/L) | 0.33 ± 0.30 | 0.35 ± 0.16 | 0.29 ± 0.01 |
| **NEU** (10^9^/L) | 1.46 ± 0.93 | 0.84 ± 0.33 | 2.79 ± 0.33 |
| **RBC** (10^12^/L) | 4.37 ± 0.32 | 4.48 ± 0.40 | 4.81 ± 0.96 |
| **HGB** (g/dL) | 9.80 ± 1.04 | 9.23 ± 0.77 | 10.47 ± 1.85 |
| **HCT** % | 24.82 ± 2.34 | 23.61 ± 2.33 | 27.39 ± 4.88 |
| **MCV** (fL) | 56.67 ± 1.45 | 52.67 ± 2.33 | 57.67 ± 1.45 |
| **MCH** (pg) | 22.27 ± 0.97 | 20.60 ± 0.38 | 21.97 ± 0.55 |
| **MCHC** (g/dL) | 39.27 ± 0.92 | 39.23 ± 1.04 | 38.17 ± 0.19 |
| **RDWs** (fL) | 50.03 ± 4.77 | 41.67 ± 1.81 | 49.47 ± 2.49 |
| **PLT** (10^9^/L) | 118.67 ± 45.16 | 145.67 ± 18.75 | 275.33 ± 80.09 |
| **MPV** (fL) | 9.07 ± 0.96 | 7.13 ± 0.28 | 7.87 ± 0.30 |
| **PDWs** (fL) | 14.80 ± 2.80 | 11.33 ± 1.71 | 12.97 ± 1.03 |
| White Blood Cell Count (WBC), Lymphocytes Count (LYM), Monocytes Count (MON), Neutrophils Count (NEU), Red Blood Cell Count (RBC), Hemoglobin Count (HGB), Hematocrit Test (HCT), Mean Corpuscular Volume Blood Test (MCV), Mean Corpuscular Hemoglobin Blood Test (MCH), Mean Corpuscular Hemoglobin Concentration Test (MCHC), Red Blood Cell Distribution Width Standard (RDWS), Platelet Count (PLT), Mean Platelet Volume (MPV), and Platelet distribution width Standard (PDWs) were analyzed by using VetScan HM5. Values reported are the mean ± SEM of 3 replicates for all the groups. | | | |

**Table S2**. Assessment of blood serum chemistry of HIV-1 infected, ART and LNP treated hu mice (n = 3).

|  | **HIV-1-infecteds and ART-treated** | | |
| --- | --- | --- | --- |
|  | **Untreated** | **C-LNP** | **T-LNP** |
| **ALB** (g/dL) | 2.10 ± 0.45 | 1.97 ± 0.82 | 1.10 ± 0.1 |
| **ALP** (U/L) | 23.33 ± 1.20 | 25.33 ± 10.87 | 17.67 ± 1.67 |
| **ALT** (U/L) | 38.33 ± 26.34 | 18.33 ± 5.84 | 12.00 ± 2.52 |
| **AMY (**U/L**)** | 568.33 ± 114.94 | 591.67 ± 170.46 | 408.67 ± 13.91 |
| **TBIL** (mg/dL) | 0.37 ± 0.03 | 0.50 ± 0.15 | 0.37 ± 0.03 |
| **BUN** (mg/dL) | 10.00 ± 4.00 | 9.00 ± 3.61 | 5.00 ± 1.15 |
| **Ca** (mg/dL) | <4.0 | <4.0 | <4.0 |
| **PHOS** (mg/dL) | 14.53 ± 1.99 | 13.40 ± 2.65 | 16.50 ± 0.36 |
| **CRE** (mg/dL) | 0.30 ± 0.01 | 0.20 ± 0.00 | <0.2 |
| **GLU** (mg/dL) | 145.33 ± 3.84 | 142.33 ± 35.26 | 122.67 ± 3.28 |
| **Na+** (mmol/L) | 165.00 ± 2.33 | 162.67 ± 2.40 | 160.67 ± 0.88 |
| **K+** (mmol/L) | >8.5 | > 8.5 | 8.05 ± 0.35 |
| **TP** (g/dL) | 3.00 ± 0.40 | 3.33 ± 1.04 | 2.37 ± 0.09 |
| **GLOB** (g/dL) | 0.90 ± 0.06 | 1.37 ± 0.23 | 1.20 ± 0.00 |
| Albumin (ALB), alkaline phosphatase (ALP), alanine transaminase (ALT), amylase (AMY), total bilirubin (TBILL), blood urea nitrogen (BUN), calcium (CA), phosphorus (PHOS), creatinine (CRE), glucose (GLU), sodium ion (Na+), potassium ion (K+), total protein (TP), and Globulin (GLOB) were analyzed by using VetScan VS2. Values reported are the mean ± SEM of 3 replicates for all the groups. | | | |
